# Magic methylation with methyl-containing peroxides

**DOI:** 10.1039/d4sc05620e

**Published:** 2024-12-04

**Authors:** Daliah Farajat, Yuhua Zhang, Chao-Jun Li

**Affiliations:** a Department of Chemistry, McGill University 801 Sherbrooke Street West Montreal Quebec H3A 2K6 Canada cj.li@mcgill.ca; b Accustandard Inc. 125 Market Street New Haven Connecticut 06513 USA; c FRQNT Centre for Green Chemistry and Catalysis Canada

## Abstract

Methyl groups rank among the most abundant carbon fragments found in natural products and small-molecule pharmaceuticals. The late-stage and environmentally friendly installation of these groups onto biologically active molecules has attracted widespread attention in both industry and academia. In 2008, we published the first use of a methyl radical derived from a peroxide toward a directed transition-metal catalysed C–H methylation. In the past sixteen years, methyl-containing peroxides have proven themselves as robust reagents for introducing methyl groups onto organic molecules. In this review, our goal is to provide a thorough summary of the research advancements achieved in this field thus far.

## Abstract 2

Methyl groups are abundant in pharmaceuticals, and their installation has garnered significant attention. This review summarises advancements in the use of methyl-containing peroxides as reagents for a variety of methylation reactions.

## Graphical abstract

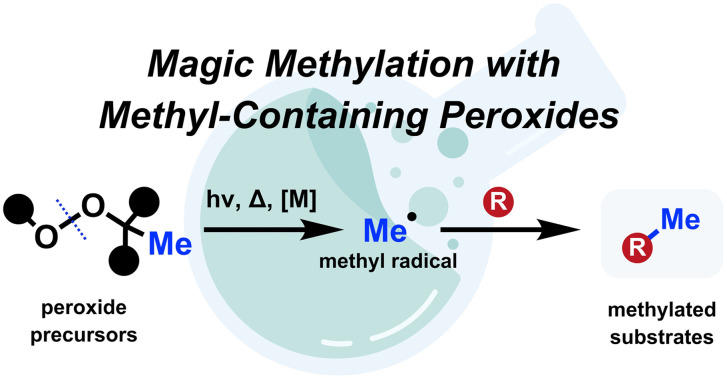

## 1 Introduction

**Scheme 1:**
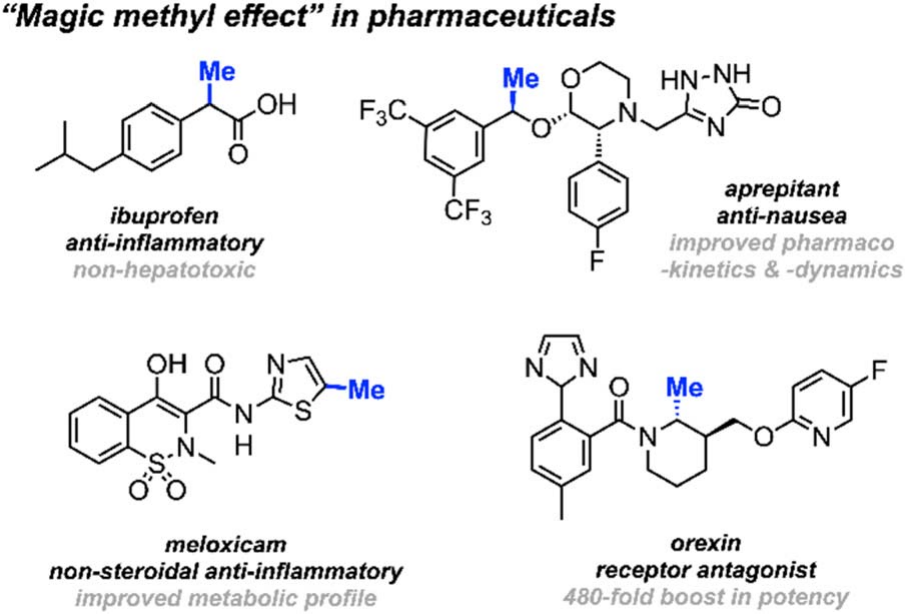
Selected examples of the “magic methyl effect” in pharmaceuticals and drug development.

Methyl groups constitute one of the most prevalent functionalities in both natural and synthetic products. Recent estimates indicate that up to 67% of the top-selling small-molecule drugs feature at least one methyl group.^1,2^ The deliberate installation of methyl groups is a common practice aimed at enhancing a molecule's bioavailability and drug–target interactions without drastically changing its molecular weight; a modification that often leads to a substantial increase in potency. This widely observed phenomenon is commonly dubbed “the magic methyl effect”.^3,4^ Consequently, methodologies involving the incorporation of methyl groups onto drug candidates attract considerable attention in the pharmaceutical industry (Scheme 1).^5^

A wide variety of methylation reactions exist, typically employing methyl-containing reagents as precursors to methyl-anions, -cations, or -radicals, depending on the specific nature of the reaction. Frequently utilised methylating reagents include methyl iodide, dimethyl sulfate, dimethyl carbonate, methyl triflate, and various methyl organometals.^6^ However, each of these reagents carries its own set of caveats, hazards, and operational challenges. Common issues include the requirement for harsh reaction conditions, as well as strong bases and stoichiometric quantities of metal additives.^7^ To date, the ongoing endeavour of discovering innovative and environmentally friendly methylating reagents persists.

**Scheme 2:**
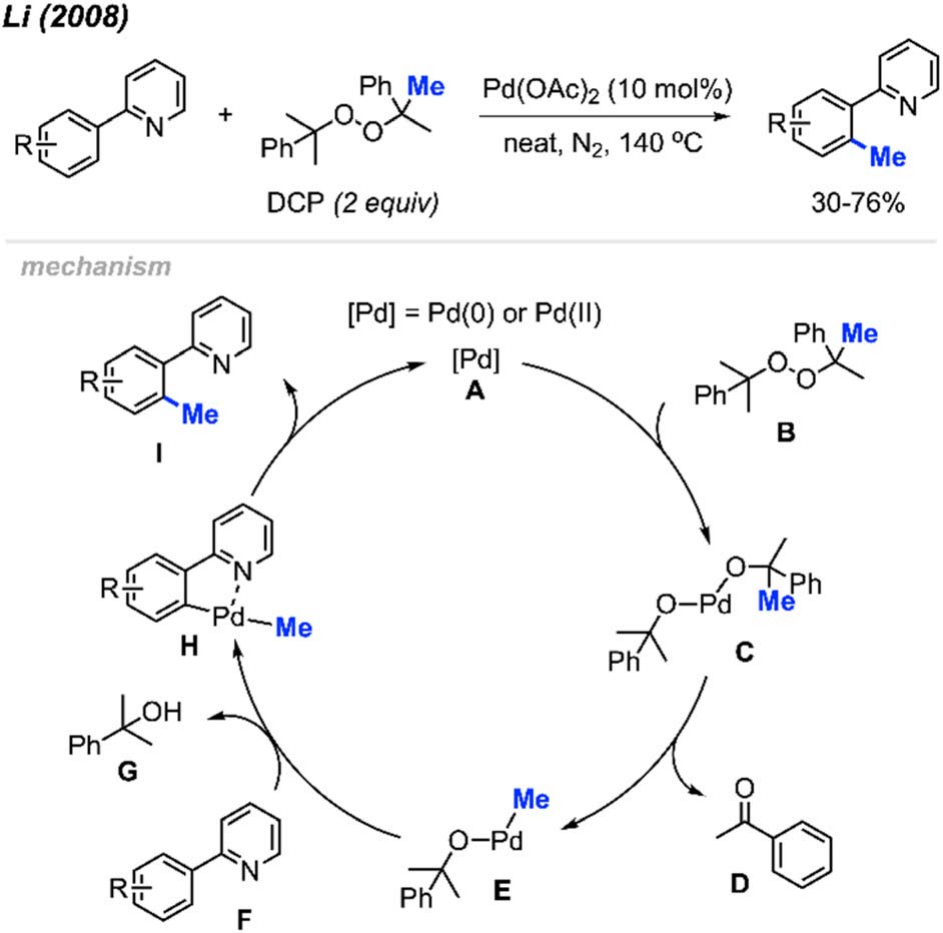
Palladium-catalysed *ortho*-methylation of 2-phenylpyridines using DCP as a methyl radical source and proposed mechanism.

For the past two decades, our research group centred its efforts on investigating transition-metal-catalysed C–H bond activation followed by C–C bond formation, namely, Cross-Dehydrogenative Couplings (CDC).^8–13^ In 2008, during an exploration of phenyl C(sp^2^)–H bond activation of 2-phenylpyridine, we remarkably discovered the formation of *o*-tolypyridine when the reaction was conducted with di-*tert*-butyl peroxide (DTBP) as an oxidant.^14^ We hypothesised that the methyl group originated from a rearrangement of DTBP radicals. Through a screening process involving various methyl-containing peroxides, dicumyl peroxide (DCP) emerged as the most effective when catalysed by Pd(OAc)_2_ (10 mol%) at 130–150 °C, yielding methylated products in the range of 30–76% (Scheme 2).

The proposed reaction mechanism involves a β-methyl elimination, generating a methyl-palladium intermediate while simultaneously releasing a ketone.^15^ Nearly stoichiometric quantities of acetophenone D and α-cumyl alcohol G were isolated by column chromatography, confirming that DCP served as not only the methylating reagent but also the hydrogen acceptor. Our publication marked the first demonstration of a transition-metal catalysed and directed methylation of a carbon–hydrogen bond using a peroxide-derived methyl radical.

**Scheme 3:**
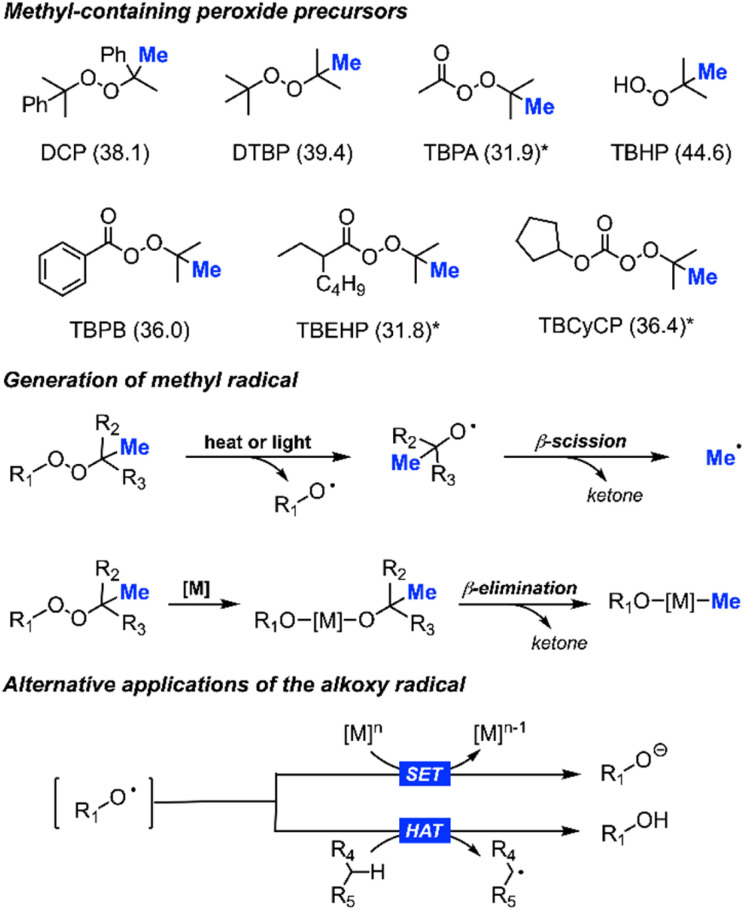
Library of peroxides used as methyl precursors (corresponding bond dissociation energy in kcal mol^−1^):^17,18^ dicumyl peroxide (DCP), di-*tert*-butylperoxide (DTBP), *tert*-butylperacetate (TBPA), *tert*-butyl hydroperoxide (TBHP), *tert*-butyl peroxybenzoate (TBPB), *tert*-butyl 2-ethylhexaneperoxoate (TBEHP), and *tert*-butyl cyclopentyl carbonoperoxoate (TBCyCP), *based on structurally similar peroxide (top); heat, light, or metal driven generation of methyl radicals (middle); alternative applications for alkoxy radicals, including single electron transfer (SET) and hydrogen atom transfer (HAT) (bottom).

Since our discovery, research groups worldwide have further studied the utilisation of various methyl-containing peroxides as powerful reagents for methylation reactions (Scheme 3). A plethora of outstanding research has been published on this chemistry, showcasing significant advancements.^16^ This has also broadened our mechanistic insights, allowing us to understand that using peroxides as methyl-containing precursors involves balancing several possible reaction pathways, including how the methyl radical is generated, single electron transfer (SET), and hydrogen atom transfer (HAT). Herein, we aim to present a comprehensive overview of the progress and developments made in this field to-date.

## 2 Direct methylation

### 2.1 Methylation of C–H bonds

Most investigations of C–H bond methylation *via* peroxides were focused on C(sp^2^)–H and C(sp^3^)–H bonds; we have yet to see any reports on C(sp)–H bonds.

#### 2.1.1 Methylation of aryl C(sp^2^)–H bonds with directing groups

##### 2.1.1.1 Palladium catalysis

**Scheme 4:**
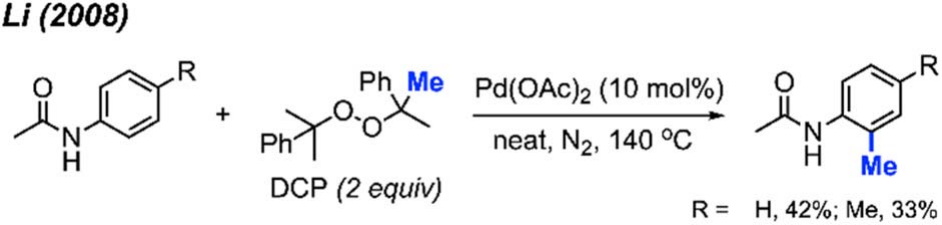
Palladium-catalysed *ortho*-methylation of anilides using DCP as a methyl source.

The Pd(OAc)_2_/DCP reaction system not only contributes to the methylation of phenylpyridine derivatives, it also promotes the C(sp^2^)–H methylation of benzenesulfonamide and benzamide compounds at the *ortho* position. In our 2008 study, we found that although acetanilide was reactive under the Pd(OAc)_2_/DCP conditions, only 33–42% yields were obtained (Scheme 4). Thus, an improved method towards methylation of acetanilide compounds by peroxide remained a desirable objective.

**Scheme 5:**
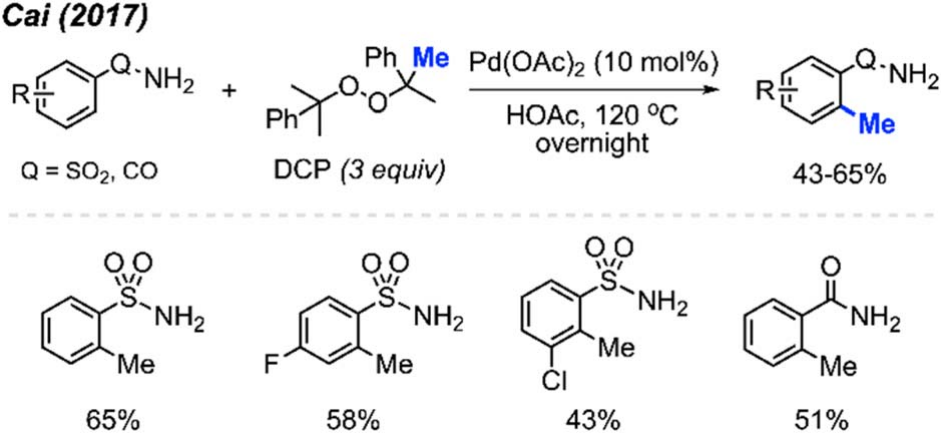
Palladium-catalysed *ortho*-methylation of benzenesulfonamide and benzamide compounds using DCP as a methyl source.

In 2017, Cai and co-workers reported that in HOAc as the solvent, Pd(OAc)_2_ (10 mol%) and DCP (3 equiv.) allowed the *ortho*-methylation of benzenesulfonamide and benzamide compounds in 43–65% yields (Scheme 5).^19^ The authors also discovered that there was no significant difference in yields whether the reaction was carried out under argon or an open air atmosphere. Although the authors only reported 4 sample results, they demonstrated progress towards *ortho*-methylation of arene compounds.

**Scheme 6:**
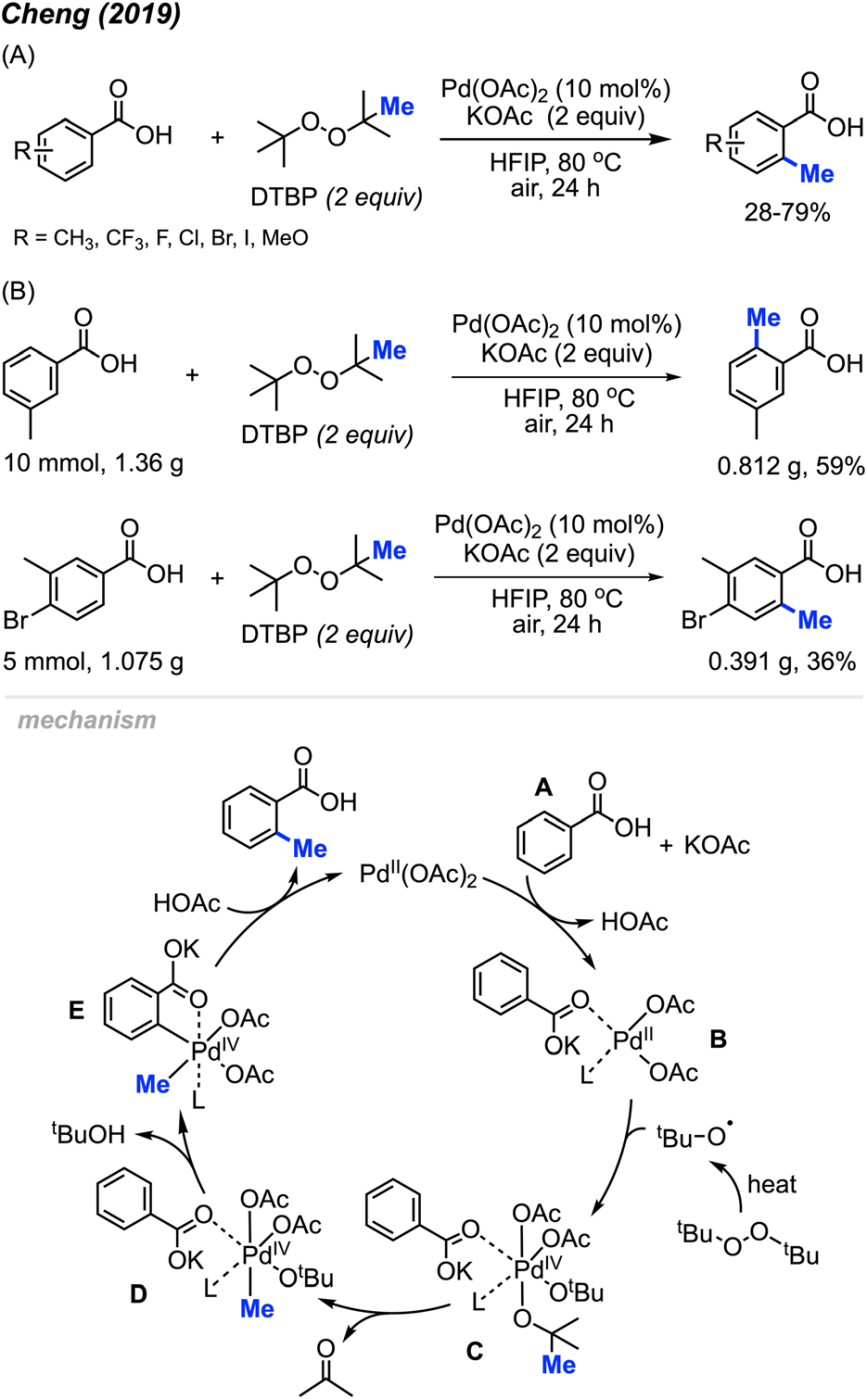
Palladium-catalysed *ortho*-methylation of benzoic acids using DTBP as methyl source. (A) Optimised reaction conditions, (B) gram scale reaction.

Aside from the aforementioned *ortho*-aryl methylation reactions using Pd(OAc)_2_/DCP reagents, the Pd(OAc)_2_/DTBP reaction system proved to be beneficial for the methylation of benzoic acids. In 2019, Cheng and co-workers reported that combining two equivalents of DTBP and potassium acetate with 10 mol% of Pd(OAc)_2_ catalysed the *ortho*-methylation of benzoic acid derivatives in 28–79% yields using 1,1,1,3,3,3-hexafluoro-2-propanol (HFIP) as a solvent heated to 80 °C (Scheme 6A).^20^ Compared to Li^14^ and Cai's^19^ reaction conditions, this reaction was milder (open to air and lower temperature), and the authors demonstrated the protocol on 18 functionally diverse samples. The use of HFIP proved to be vital, as the reaction does not proceed without it. Various functional groups (methyl, trifluoromethyl, halogen, and methoxy) at *meta*-, *ortho*- and *para*-positions on benzoic acid, as well as disubstituted benzoic acids are well tolerated in this reaction. Notably, the *meta*-positioned substituent directs the methylation selectively towards the less hindered activated C–H bond. It is also worth pointing out that 54% and 34% isolated yields were obtained for gram scale reactions (Scheme 6B), which demonstrated the potential of this methylation reaction towards practical applications.

When 2,2,6,6-tetramethylpiperidine-1-oxyl (TEMPO) was added, the reaction was completely inhibited, which indicated the involvement of a radical process. As shown in Scheme 5, Pd(OAc)_2_ reacts with benzoic acid A in the presence of KOAc to form Pd(ii) species B. The *tert*-butoxy radical generated from DTBP under heat then reacts with intermediate B to yield intermediate C. Next, β-methyl elimination of species C forms intermediate D. Subsequently, Pd(iv) D undergoes C–H insertion to generate intermediate E and *t*-BuOH. Finally, reductive elimination of species E produces the *ortho*-methylation product and regenerates Pd(OAc)_2_.

##### 2.1.1.2 Cobalt catalysis

**Scheme 7:**
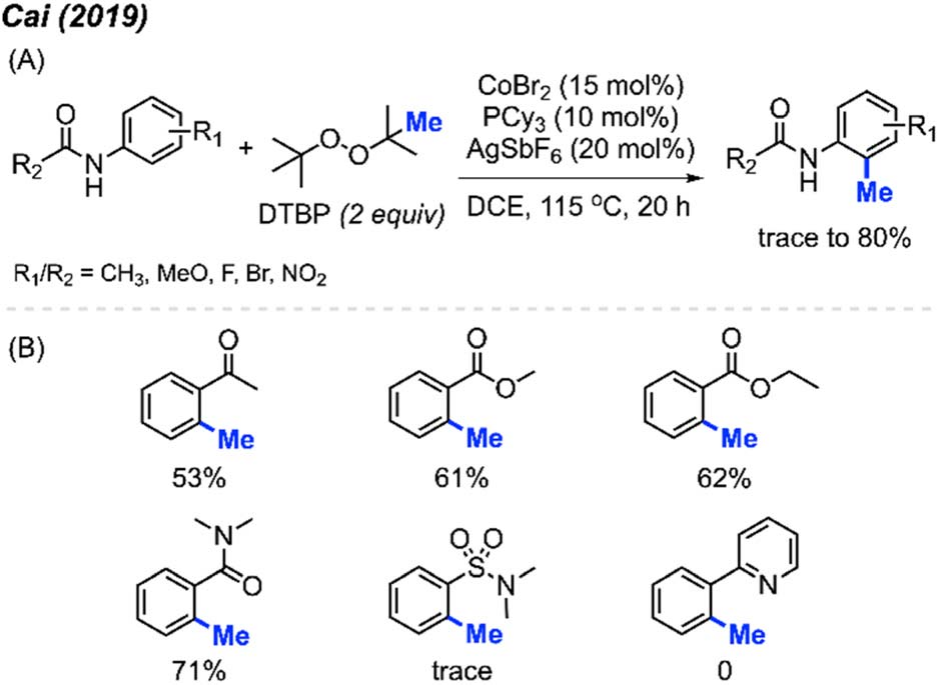
Cobalt-catalysed *ortho*-methylation of anilides and arenes using DCP as a methyl source. (A) Optimised reaction conditions, (B) reaction yields with different directing groups.

In 2019, Cai and co-workers reported the successful *ortho*-methylation of anilides by using a cobalt/silver catalyst system in the presence of DTBP (2 equiv.). The reaction produced up to 80% yield and a total of 16 methylated anilide derivatives were reported (Scheme 7A).^21^ It was found that the arenes containing directing groups such as amides, ketones and esters also gave good yields of the methylated products. Interestingly, this rection method did not work with 2-phenylpyridine compounds at all (Scheme 7B). Although the Pd(OAc)_2_/DCP system reported by Cai in 2017 allowed the *ortho*-methylation of benzamides, only two examples were provided (Schemes 5 and 7). In the previous examples, the *ortho*-methylation of benzamide compounds varied depending on the nature of the *N*-substituent.

**Scheme 8:**
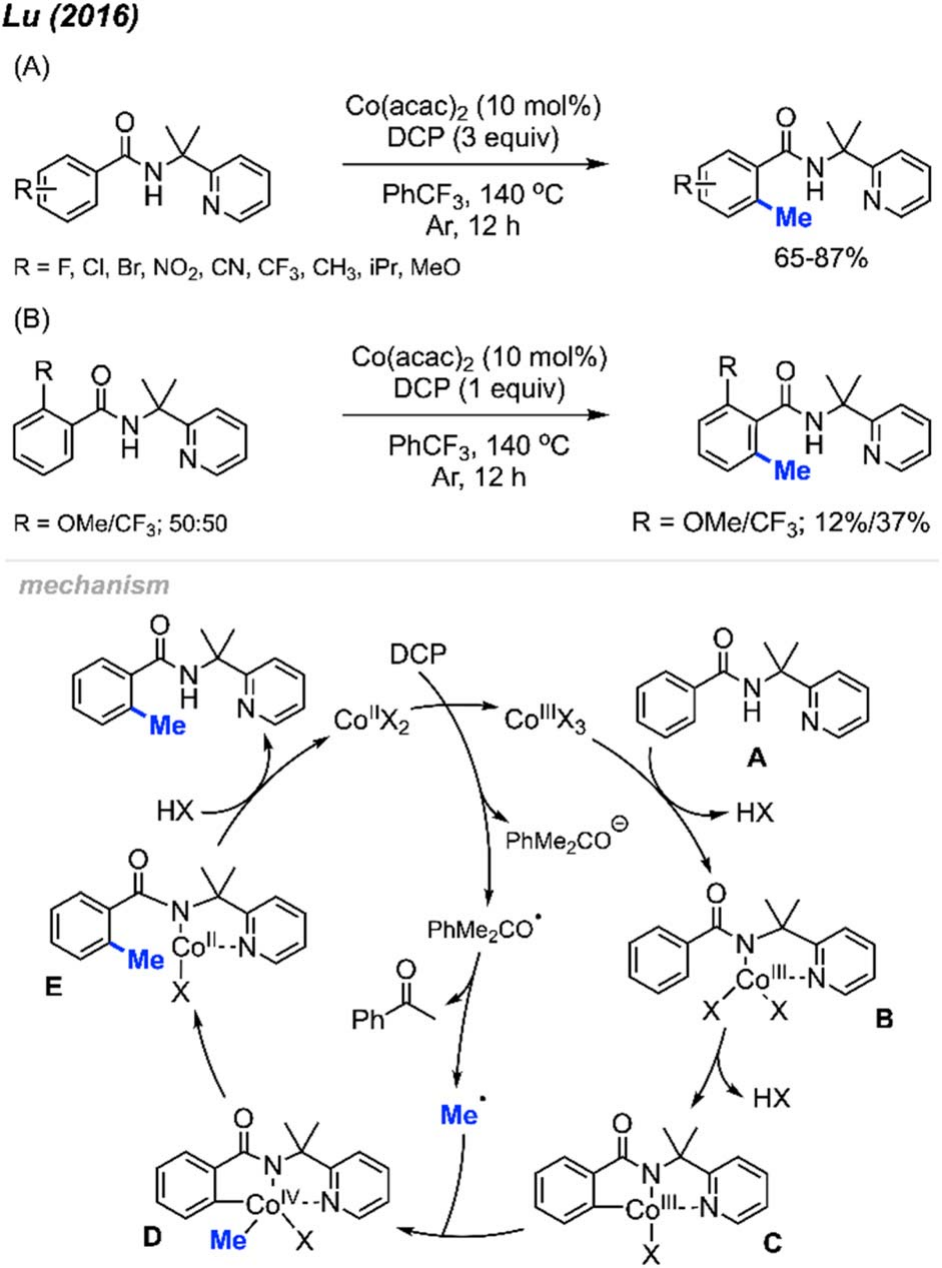
Cobalt-catalysed and directing group facilitated *ortho*-methylation of benzamide using DCP as a methyl source. (A) Optimised reaction conditions, (B) intermolecular competition experiment.

In 2016, Lu and co-workers reported an *ortho*-methylation of benzamide bearing a 2-pyridinylisopropyl (PIP) directing group, which was catalysed by 10 mol% Co(acac)_2_ with DCP (3 equiv.) (Scheme 8A).^22^ A total of 17 substrates were reported, and the isolated yields ranged between 65–87%. When the *meta*-position was occupied, only the mono-methylated product was obtained, which is in agreement with Cheng's observation^20^ (Scheme 6). The authors also performed an intermolecular competition experiment, showing that an electron-withdrawing group (–CF_3_) on the aromatic ring favours substrate methylation compared to an electron-donating group (–OMe) (Scheme 8B). Notably, the authors successfully methylated heteroaryl derivatives (thiophenes) and included one example of a cyclic α,β-unsaturated amide.

A possible mechanism is shown in Scheme 8. Upon heating, DCP undergoes a single electron transfer (SET) process with Co(ii), which forms the Co(iii) species along with one radical and one anionic alkoxy fragments of DCP. The Co(iii) species further coordinates to the benzamide molecule A by ligand exchange to form intermediate B. Then intermediate B undergoes C–H insertion to generate Co(iii) species C. β-Methyl elimination within the DCP alkoxy radical fragment generates a methyl radical, which then reacts with intermediate C to yield species D by oxidative addition. Finally, reductive elimination within species D and subsequent protonation yields the final product and regenerates the Co(ii) catalyst.

##### 2.1.1.3 Nickel catalysis

**Scheme 9:**
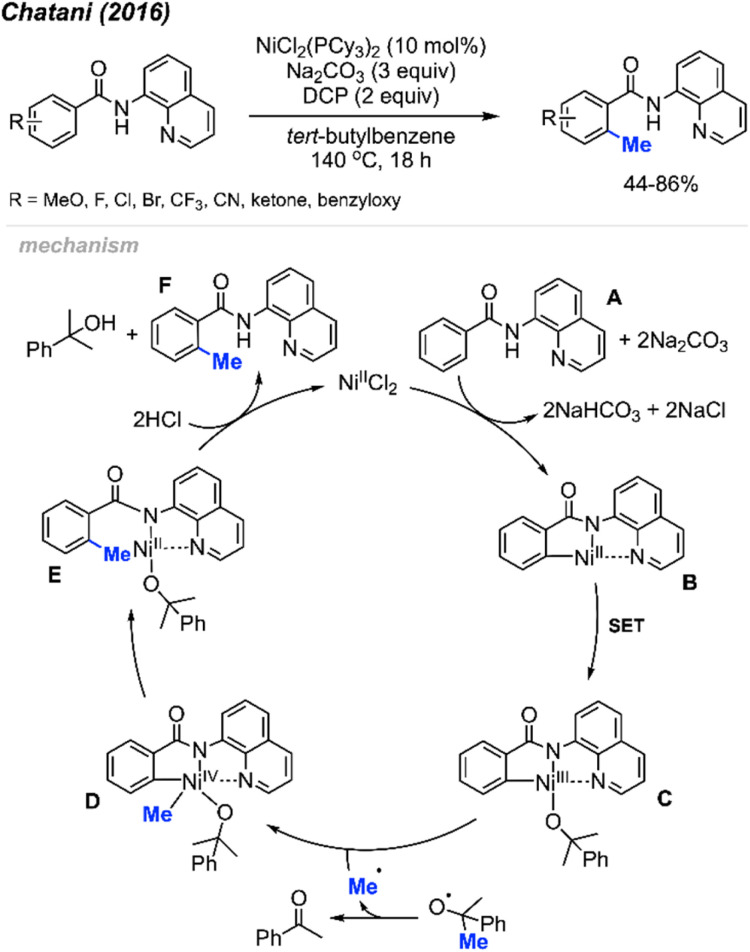
Nickel-catalysed *ortho*-methylation of benzamides containing 8-aminoquinoline directing groups and using DCP as a methyl source.

In the same year (2016), Chatani and co-workers reported the *ortho*-methylation of benzamides containing 8-aminoquinoline as a directing group. Products were isolated in 44–86% yields in the presence of 10 mol% NiCl_2_(PCy_3_)_2_, DCP (2 equiv.) and Na_2_CO_3_ (3 equiv.) using *tert*-butylbenzene as the solvent (Scheme 9).^23^ The scope included 30 benzamide derivatives with various functional groups. The authors found that *meta*-substituted benzamides favour methylation at the less sterically hindered C(sp^2^)–H bond, which agrees with the discovery by both Cheng^20^ (Scheme 6) and Lu^22^ (Scheme 8). Through a competition experiment, the authors demonstrated that electron withdrawing groups on the aromatic ring promote the methylation reaction compared to electron donating groups, which aligns with Lu's^22^ investigation in 2016 (Scheme 8).

The authors performed detailed mechanistic studies and proposed a Ni(ii)/Ni(iv) catalytic cycle (Scheme 9). First, the benzamide A reacts with the Ni(ii) species with the help of Na_2_CO_3_ base to form intermediate B. Then, a SET process between DCP and Ni(ii) B generates the Ni(iii) specie C and an alkoxy radical fragment. The alkoxy radical will decompose to yield acetophenone and a methyl radical which will react with species C to form Ni(iv) D. Finally, intermediate D undergoes reductive elimination to give intermediate E, and subsequent protonation yields the target product F and regenerates the Ni(ii) catalytic species.

**Scheme 10:**
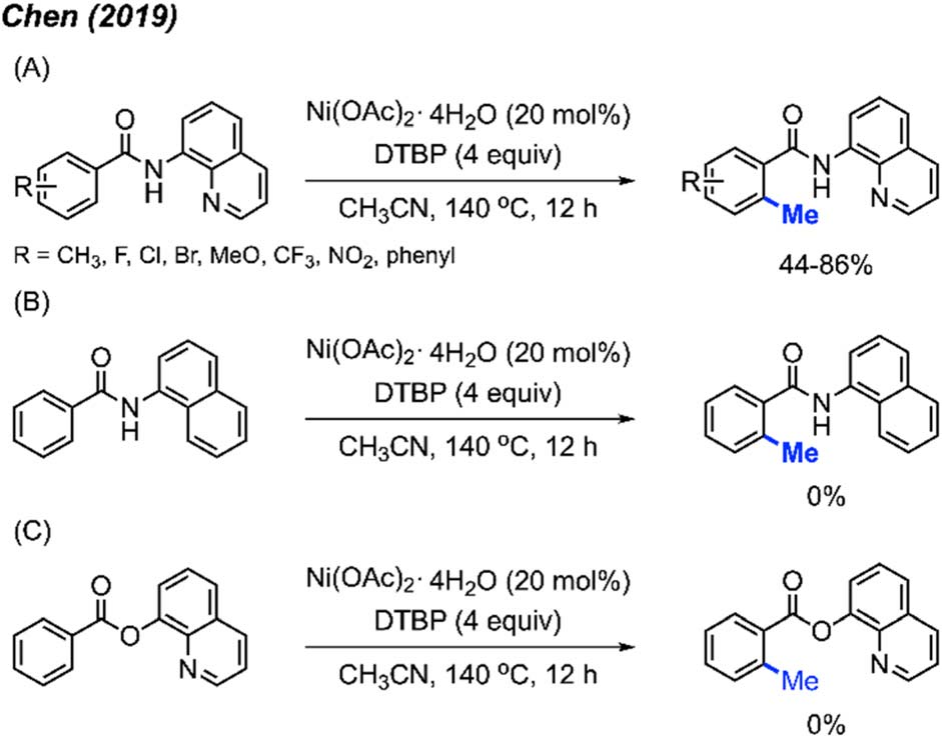
Nickel-catalysed methylation of benzamides using DTBP as a methyl course. (A) Optimised reaction conditions, (B) replacing quinoline with naphthalene, (C) replacing amide with carboxylic ester.

In 2019, Chen and co-workers reported an alternative catalytic system for benzamide methylation, with 20 examples being reported.^24^ They found that benzamide compounds can be *ortho*-methylated in the presence of Ni(OAc)_2_·4H_2_O (20 mol%) and DTBP (4 equiv.) in CH_3_CN at 140 °C for 12 hours, in 44–81% yields (Scheme 10A). Compared to the Chatani^23^ method (Scheme 9), this reaction eliminates the need for added base and generates acetone as a by-product which can more readily be removed compared to acetophenone.

In agreement with previous observations, the authors also found that *meta*-substituted benzamides encouraged methylation at the less sterically hindered site, and that electron-withdrawn groups on the aromatic ring are favoured for this reaction. Furthermore, the authors demonstrated that both the quinoline and amide components were necessary functionalities within the directing group. If the quinoline is replaced with a naphthalene, or if the amide is replaced with a carboxylic ester, no product is observed in either scenario (Scheme 10B and C).

#### 2.1.2 Methylation of heteroaryl and heterocyclic C(sp^2^)–H bonds

Alongside numerous studies on the methylation of aryl C(sp^2^)–H bonds, much focus has been placed on methylating heteroaryl and heterocyclic compounds as well.

##### 2.1.2.1 Photocatalysis

**Scheme 11:**
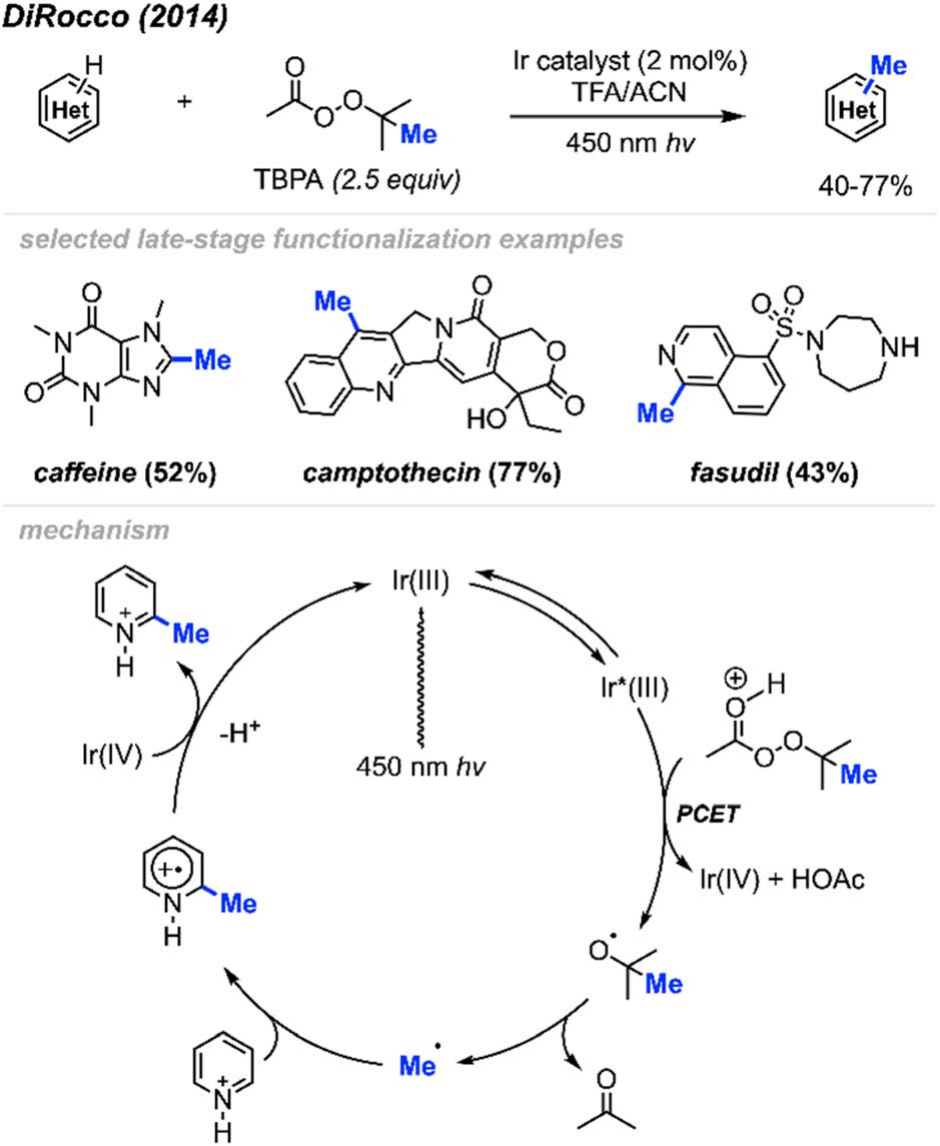
Bio-active heterocycle methylation through photoredox catalysis using TBPA.

In 2014, DiRocco and co-workers reported the late-stage photoredox catalysed methylation of biologically active heterocycles (Scheme 11).^25^ Six-membered aromatic heterocycles (*e.g.* pyridines, pyrazines, and pyrimidines) and some electron-rich five-membered aromatic heterocycles (*e.g.* imidazoles) reacted with peroxides efficiently. Notably, this reaction proceeds in the presence of various functionalities such as basic amines, alcohols, amides, and esters without the need for protecting groups. The author also found that DTBP and TBHP did not work for this reaction, with *tert*-butylperacetate (TBPA) being the best methylation reagent.

The proposed catalytic cycle is shown in Scheme 11. A proton-coupled electron transfer (PCET) between the excited Ir(iii)* photoredox catalyst and TBPA under acidic conditions generates a *tert*-butoxy radical, which subsequently decomposes *via* β-scission, yielding a methyl radical and acetone. The methyl radical then undergoes addition to the protonated heterocycle, resulting in an amino-radical cation, which is then oxidised by Ir(iv), yielding the desired product and regenerating the initial Ir(iii) photocatalyst.

##### 2.1.2.2 Metal-free, thermal reactions

**Scheme 12:**
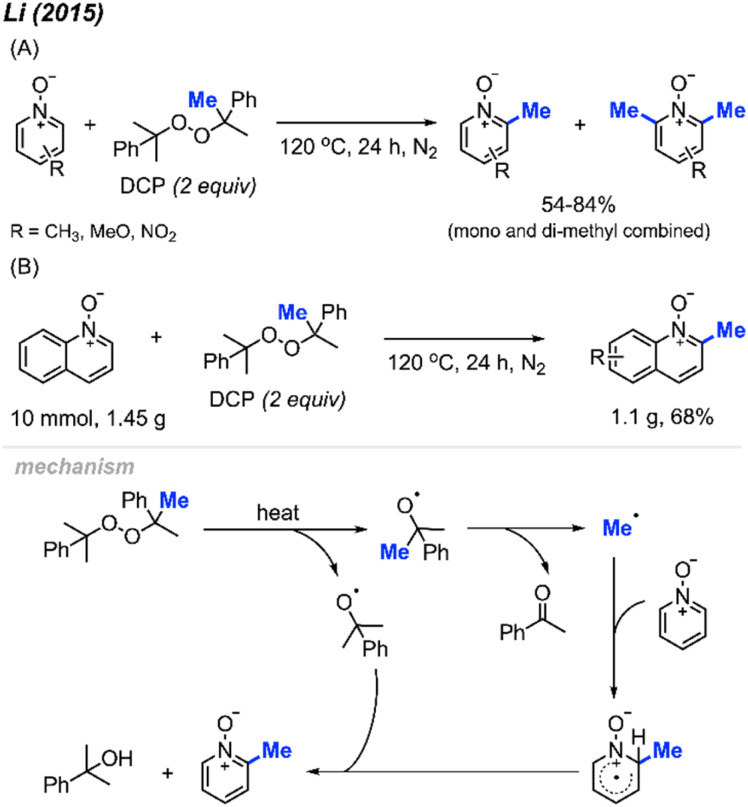
Methylation of pyridine *N*-oxide derivatives with DCP. (A) Optimised reaction conditions, (B) gram scale reaction.

In 2015, Li and co-workers reported that DCP can be used for methylation of pyridine *N*-oxides at the 2-position under inert atmosphere at 120 °C in the absence of a metal catalyst.^26^ The authors provided 13 examples containing various functionalities (Cl, NO_2_, and OCH_3_), with yields ranging from 54–84% (Scheme 12A). It is noted that earlier studies show that methyl radicals from high temperature decomposition of peroxides can generate trace and mixture of methylation products with heterocycles.^27,28^ Not only were pyridine *N*-oxides effective substrates under these conditions, methylation of quinoline *N*-oxide compounds and isoquinoline *N*-oxide also gave good yields. Furthermore, the authors performed a gram scale reaction, and were able to obtain a 68% isolated yield (Scheme 12B). Similar to our work in 2008, this pyridine *N*-oxide methylation method suffers from the generation of both mono- and di-methylated products, which can complicate purifications. This study significantly extended the radical methylation *via* a peroxide that did not require transition metal catalysis.

The reaction mechanism (Scheme 12) begins with DCP decomposition under heat, generating two molecules of 2-phenylpropanoxy radical, which can further undergo β-scission to form the methyl radical and acetophenone. The methyl radical then attacks the *N*-oxide at the 2-position, forming an intermediate amino-radical cation. With the help of another DCP derived radical, hydrogen abstraction occurs to restore the aromatic ring.

**Scheme 13:**
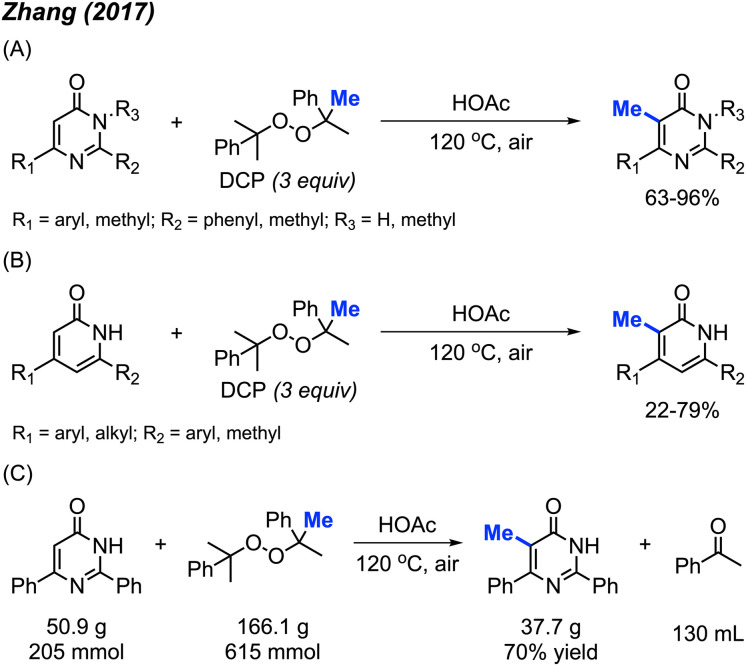
Metal-free methylation of pyrimidinones and pyridinones using DCP. (A) Optimised reaction conditions for pyrimidinones, (B) optimised reaction conditions for pyridinones, (C) gram scale reaction.

In 2017, Zhang and co-workers discovered that metal-free conditions can be used to methylate pyrimidinones and pyridinones in acetic acid with DCP (3 equiv.) at 120 °C.^29^ 14 examples of pyrimidinones were provided with 63–96% yields (Scheme 13A) and 18 examples of pyridinones were generated in 22–79% yields (Scheme 13B). For both pyrimidinone and pyridinones compounds, it was observed that aromatic substituents improved yields compared to non-aryl groups and that compounds lacking at least one aryl substituent were unreactive. This observation can be attributed to the ability of aromatic groups to better stabilise the intermediate radical formation. Notably, there is one example of the C-3 methylation of indole, albeit at a low yield of 22%.

The authors also performed a 50 g scale reaction, with 70% isolated yield being achieved (Scheme 13C), which indicated its utility in organic synthesis. The proposed mechanism is in-line with previously reported radical mechanisms (Scheme 12). First, homolysis followed by β-scission of DCP generates the methyl radical and acetophenone by-product. This is followed by selective addition of the methyl radical at the 5-position, and subsequent re-aromatisation to generate the final product.

**Scheme 14:**
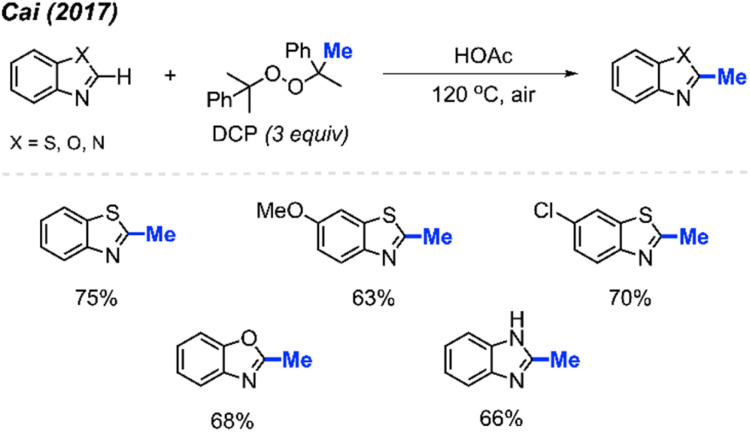
Metal-free methylation of azoles using DCP.

By using the exact same reaction conditions as Zhang^29^*et al.* (Scheme 13), Cai and co-workers reported the direct methylation of azole compounds in up to 75% yields, with 5 examples being reported (Scheme 14).^30^ This work further highlighted the versatility of peroxide-mediated methylation and broadened the scope of valuable heterocycles that it can be applied to.

**Scheme 15:**
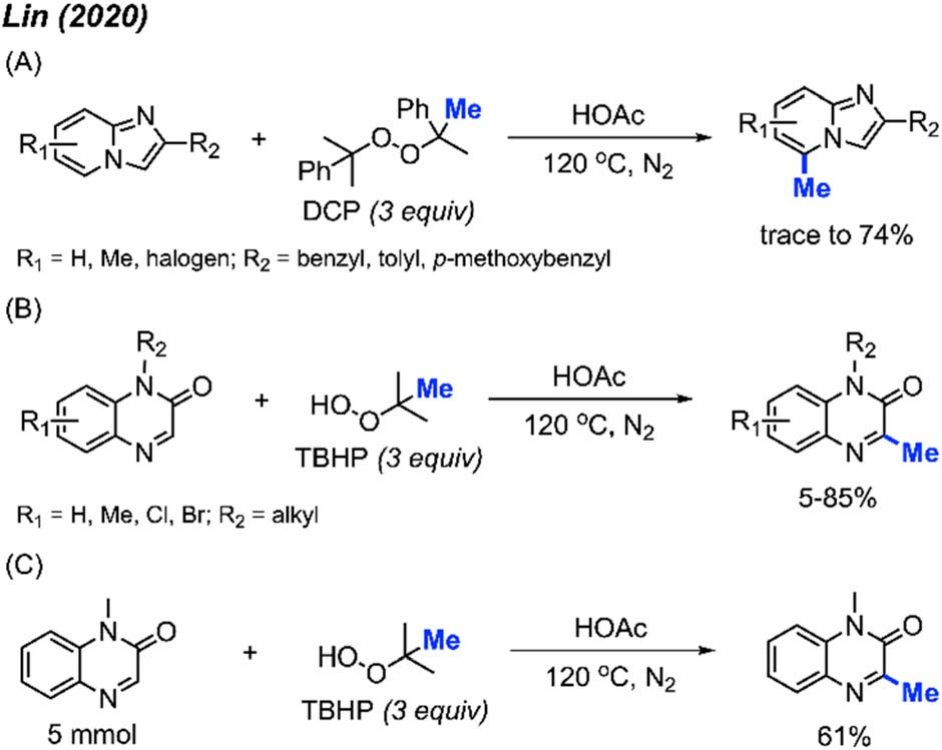
Metal-free methylation of imidazo[1,2-*a*]pyridines and quinoxalin-2(1*H*)-ones using either DCP or TBHP. (A) Optimised reaction conditions for imidazo[1,2-*a*]pyridine, (B) optimised reaction conditions for quinoxaline-2(1*H*)-one, (C) gram scale reaction.

In 2020, Lin and co-workers discovered a third application of the same metal-free DCP-mediated methylation of imidazo[1,2-*a*]pyridine compounds at the C-5 position (Scheme 15A).^31^ A total of 11 imidazopyridine compounds were methylated, with yields ranging from trace amounts to 74%. Both electron-donating groups (methyl, methoxy) and electron-withdrawing groups (halogen) were tolerated, however, no specific trends in reactivity were observed based on substituent variation.

Although previous investigations into the use of *tert*-butyl hydroperoxide (TBHP) as a methylating reagent were not successful, the authors found that TBHP works excellently for the C-3 methylation of quinoxaline-2(1*H*)-one compounds. Under the same metal-free conditions, TBHP was shown to be the most suitable reagent compared to the commonly used DCP and DTBP (Scheme 15B). 15 examples were provided in trace-85% yields. A gram scale reaction gave 61% isolated yield (Scheme 15C).

**Scheme 16:**
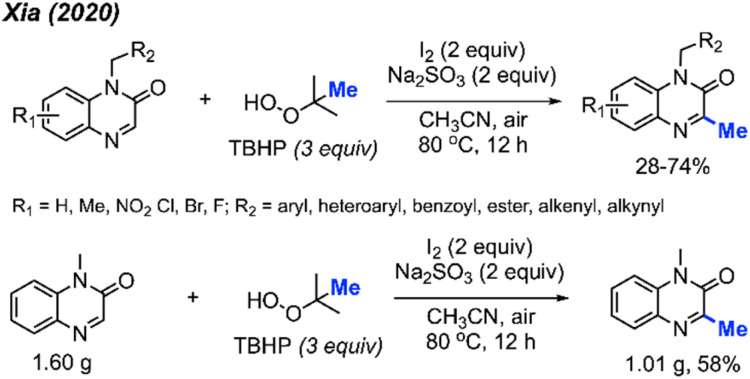
Metal-free methylation quinoxaline-2(1*H*)-ones using TBHP.

In the same year, Xia and co-workers also developed a method for C-3 methylation of quinoxaline-2(1*H*)-one compounds (Scheme 16).^32^ In this case, the iodine-mediated methylation was achieved at a lower temperature of 80 °C in air, using TBHP as the methyl source. The authors report a large scope with yields ranging from 28–74%, with good functional group tolerance including aliphatic chains, esters, alkenes, alkynes, methoxy, nitro and halogens.

##### 2.1.2.3 Copper catalysis

**Scheme 17:**
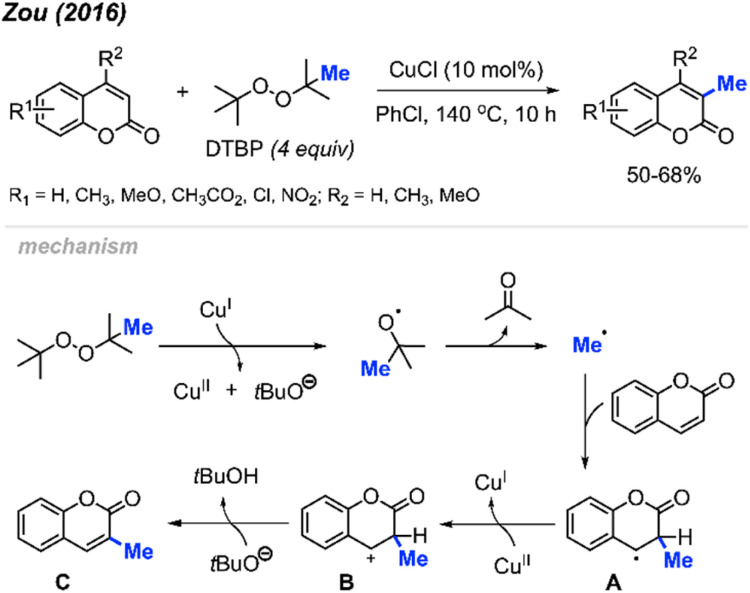
Copper-catalysed regioselective methylation of coumarins.

In 2016, Zou and co-workers reported the methylation of coumarins catalysed by Cu(i) using DTBP as the methylation reagent (Scheme 17).^33^ The authors methylated 17 variously functionalised coumarins, which provided 50–68% yields. Higher yields were observed when electron-withdrawing groups were present on the phenyl ring compared to electron-donating groups, which likely increased the electrophilicity at the 3-position. Interestingly, the lower yields observed with electron-donating groups on the phenyl ring can be improved with the addition of an electron-donating groups at the 4-position, likely due to the compensatory stabilisation of the benzyl radical intermediate.

The authors proposed a mechanism (Scheme 17) that begins with Cu(i)-mediated cleavage of DTBP to generate a *tert*-butoxyl radical, which then undergoes β-scission to generate the methyl radical and a molecule of acetone. Selective addition of the methyl radical to the 3-position of coumarin forms the benzyl radical intermediate A, which is then oxidised by Cu(ii) to yield the carbocation B. Finally, deprotonation of intermediate B produces product C.

##### 2.1.2.4 Iron catalysis

**Scheme 18:**
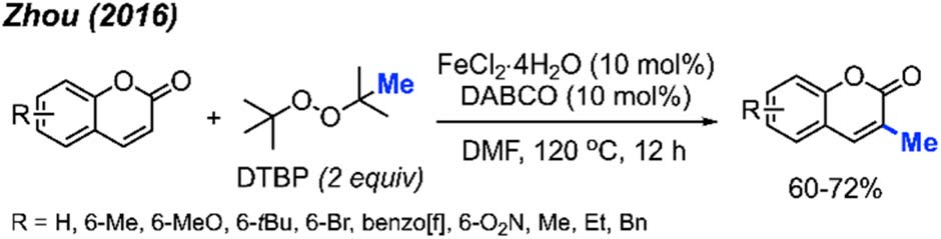
Iron-catalysed C-3 methylation of coumarins using DTBP.

Again in 2016, Zhou and co-workers reported an alternative method for C-3 methylation of coumarins, catalysed by FeCl_2_·4H_2_O (10 mol%) in the presence of DABCO (10 mol%) base and DTBP as a methyl radical source.^34^ This is the second example of coumarin methylation by DTBP, generating yields from 60–72% for 7 coumarin derivatives (Scheme 18).

**Scheme 19:**
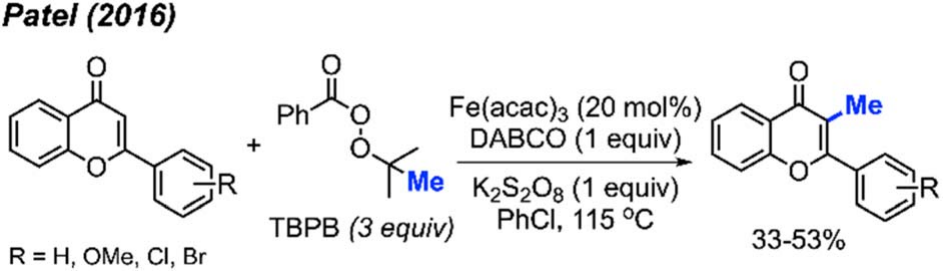
Iron-catalysed C-3 methylation of flavones with TBPB.

In the same year, Patel and co-workers discovered that *tert*-butyl peroxybenzoate (TBPB) functions as a reagent for the C-3 methylation of flavones when catalysed by Fe(acac)_3_ (Scheme 19).^35^ Although their publication focused on the use of TBPB as an intermediate oxidant for C-3 cyclo-alkylation and amidation, 5 examples of flavone methylation were provided using TBPB as the methyl source. Despite moderate to low yields, these examples highlight the ability of peroxide-derived methyl radicals to functionalise electron-rich sites of heterocycles.

#### 2.1.3 Methylation of alkenyl C(sp^2^)–H bonds

Research on the direct methylation of alkenyl C(sp^2^)–H bonds is far less extensive, however, there have been some important developments in this area in recent years. To-date, these advances have been focused on styrene derivatives.

**Scheme 20:**
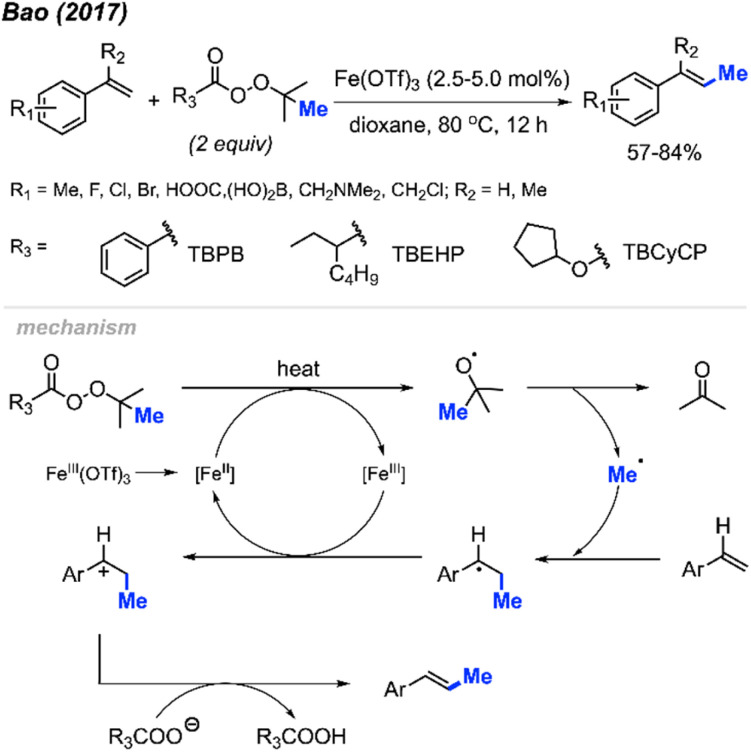
Iron-catalysed direct C–H methylation of vinyl arenes.

The first report of a direct alkenyl C(sp^2^)–H methylation was published by Bao and co-workers in 2017, where they demonstrated an iron-catalysed methylation of vinyl arenes (Scheme 20).^36^ Using 2.5–5 mol% Fe(OTf)_3_ in dioxane at 80 °C, the authors achieved yields ranging from 57–84% using a variety of methyl-containing peroxides. Functional group tolerance included methyl groups, halogens, carboxylic acid, and dihydroxyborate.

The authors proposed the mechanism shown in Scheme 20, starting with the initial reduction of Fe(OTf)_3_ to Fe(ii). The heat along with Fe(ii) then facilitates the decomposition of the peroxide to form the methyl radical, a carboxylate and acetone byproduct. The methyl radical will then react at the terminal end of the alkene, forming the more stable benzylic radical that is subsequently oxidised to the carbocation by Fe(iii) thereby reforming the Fe(ii). A final deprotonation of the substrate by the carboxylate allows for the reformation of the double bond to form the final product.

**Scheme 21:**
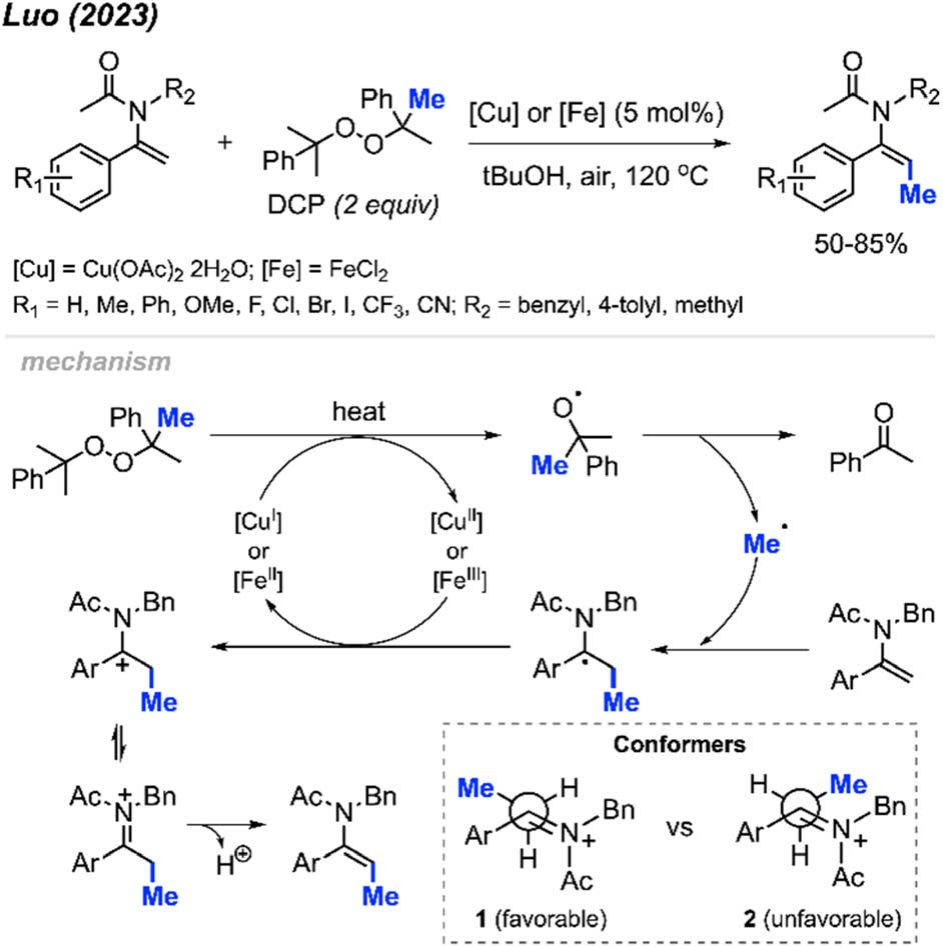
Copper- or iron-catalysed methylation of enamides using DCP.

In 2023, Luo and co-workers discovered that the methylation of enamide compounds using DCP can be copper- or iron-catalysed (Scheme 21).^37^ Both Cu(OAc)_2_·2H_2_O (5 mol%) and FeCl_2_ (5 mol%) can catalyse the direct β-C(sp^2^)–H methylation of enamides in 50–85% yields, with only *E*-isomer formation. It was found that the α-C(sp^2^) position needed to be aromatic in order to stabilise the intermediate radical generated, since aliphatic substitution at this position did not work under these conditions. Various functional groups were tolerated on the α-aryl moiety, where electron-donating groups generated slightly higher yields compared to electron-withdrawing groups.

The authors proposed a mechanism to explain the exclusive *E*-isomer product formation (Scheme 21). As with previous mechanisms, homolytic cleavage of DCP followed by β-scission generates a methyl radical and acetophenone by-product. Regioselective addition of the methyl radical at the β-alkene position is favoured by the more stable radical intermediate formed. Either Cu(ii) or Fe(ii) then oxidises the radical to form a cationic intermediate or iminium ion. A look at the possible conformers shows that conformer 1 is sterically more favourable than conformer 2. Finally, deprotonation of conformer 1 generates the *E*-configured enamide product.

#### 2.1.4 Methylation of C(sp^3^)–H bonds

The research on C(sp^3^)–H methylation *via* peroxides is far less extensive compared to C(sp^2^)–H, and only methylation at the methylene C(sp^3^)–H bond has good results.

**Scheme 22:**
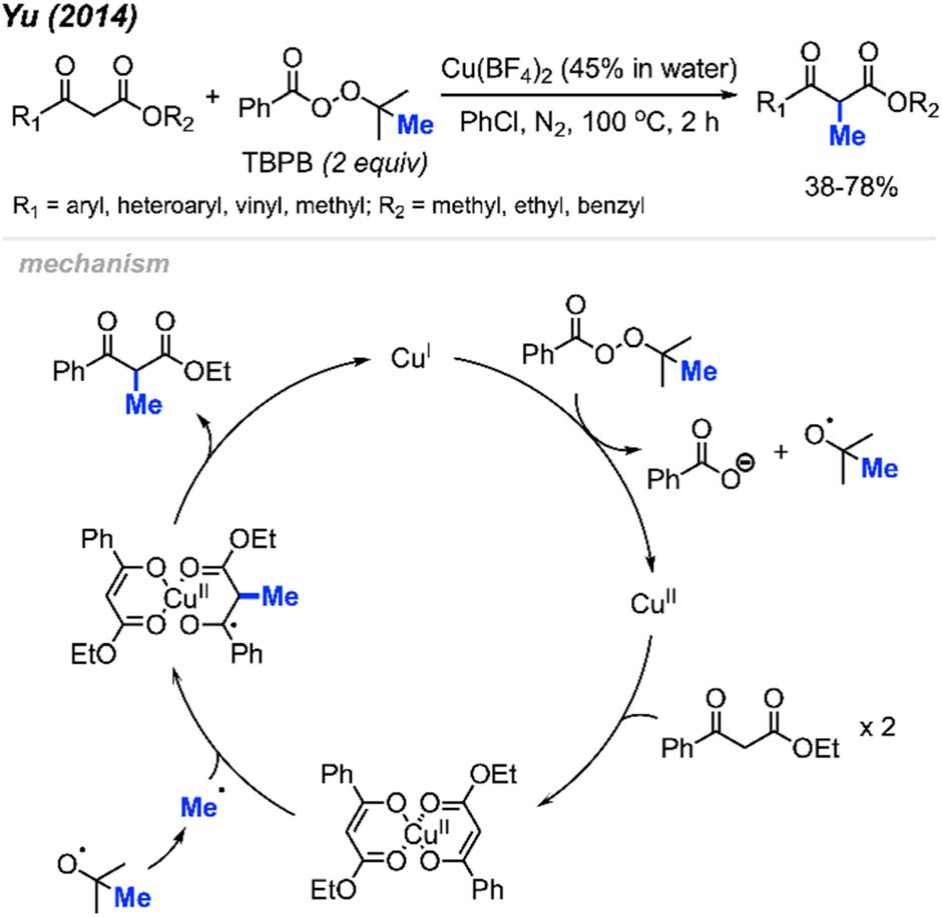
Copper-catalysed methylation of β-keto esters using TBPB.

In 2014, Yu and co-workers reported that *tert*-butyl peroxybenzoate (TBPB) could promote α-methylation of β-keto ester compounds (Scheme 22).^38^ They found that Cu(BF_4_)_2_ (5 mol%) with TBPB (2 equiv.) in PhCl as a solvent were the best conditions for methylation of 18 examples in 38–78% yields. Notably, α-methylation of dimedone was achieved at 49% isolated yield using this method, which hints at the versatility of this method.

The authors proposed the mechanism shown in Scheme 22. At 100 °C, TBPB undergoes homolytic cleavage followed by β-scission to form the active methyl radical and one equivalent of acetone. Concurrently, the 1,3-dicarbonyl compound coordinates with Cu(ii) to produce the copper-enolate intermediate. This intermediate reacts with the methyl radical to form the α-methylated 1,3-dicarbonyl product and Cu(i), which oxidises to regenerate Cu(ii) by TBPB.

**Scheme 23:**
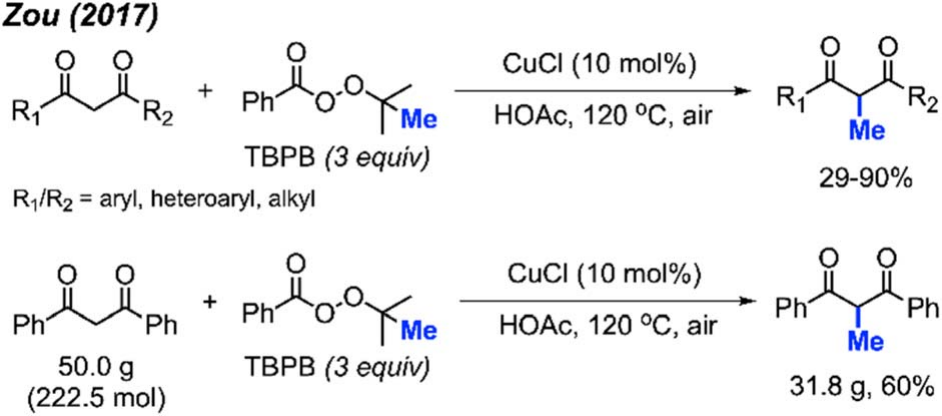
Copper-catalysed methylation of 1,3-diketones using TBPB.

In 2017, Zou and co-workers found that CuCl (10 mol%) with TBPD (3 equiv.) in HOAc as a solvent can methylate 1,3-diketone compounds at the methylene position when heated to 120 °C (Scheme 23).^39^ The authors reported 32 product examples with yields ranging from 29–90%. Under these reaction conditions, methylation of dimedone was achieved in 68% yield, much better than the method in Scheme 22.

Aromatic and aliphatic substituted as well as cyclic 1,3-diketones all reacted with TBPD to give moderate to good yields. The authors found that electronic effects are important in the reaction: 1,3-diketones with electron-withdrawing halogens and nitro groups on the phenyl rings gave lower yields, while diketones with electron-donating groups such as methoxy on the phenyl ring achieved higher yields. Various heteroaryl (pyridine, quinoline, pyrrole, thiophene, and furan) substituted 1,3-diketone also generated moderate to good yields. The aromatic-aliphatic asymmetric diketones also worked very well, however, ethyl benzoylacetate was not reactive under these conditions, which contrasts with the previously reported method by Yu *et al.* Notably, the pyridine and quinoline substituted diketones afforded demethylation as minor by-products. In the case of pyrrole and furan, *N*-methylation of pyrrole and 5-methylation of furan in 19% and 29% yields respectively were obtained. The reaction was also successfully scaled up to 50 grams, yielding 60% isolated product (Scheme 23).

**Scheme 24:**
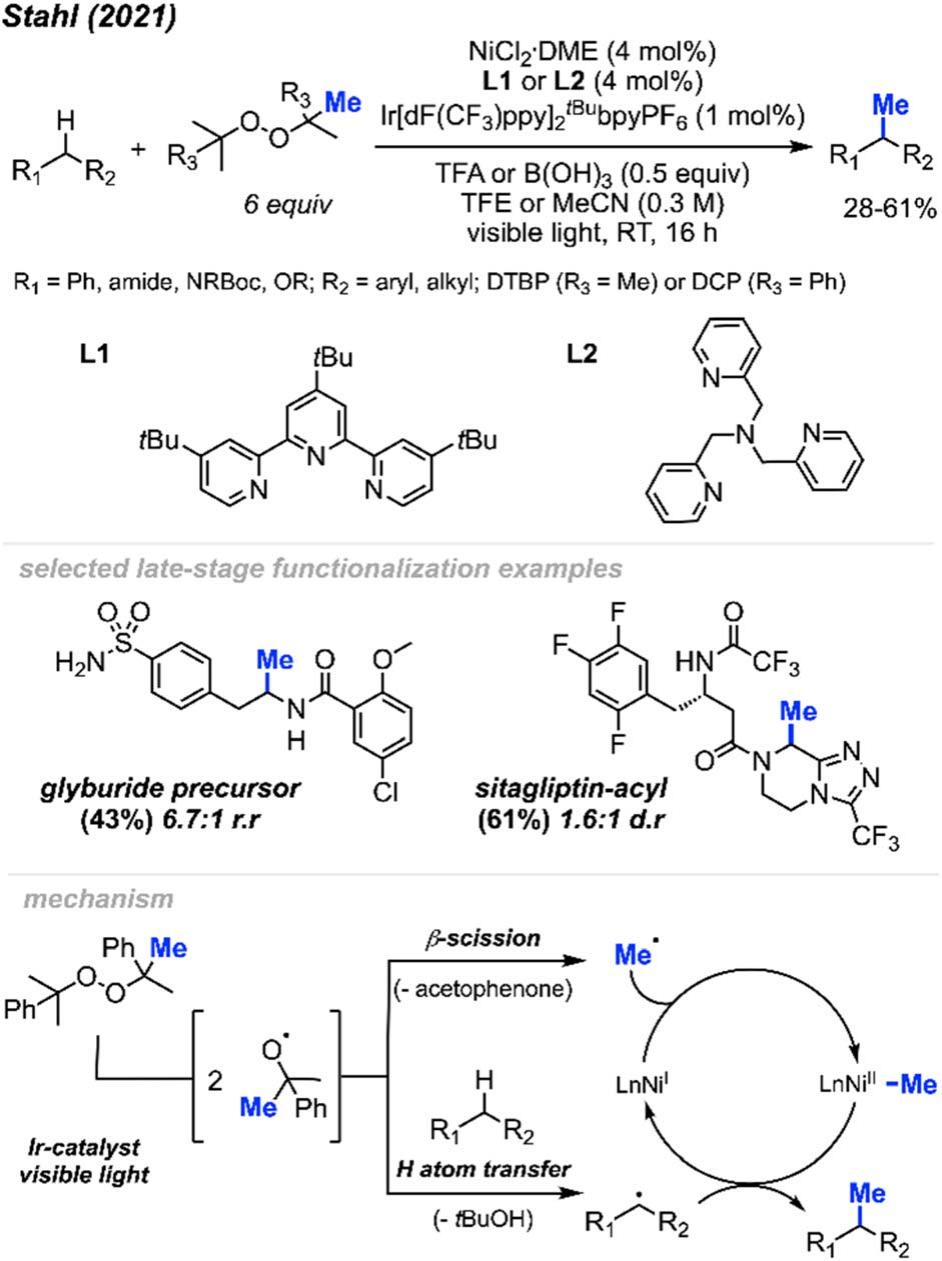
Nickel-mediated C(sp^3^)–H methylation under various conditions.

In 2021, Stahl and colleagues introduced a single-step process for methylating C(sp^3^)–H bonds employing nickel photocatalysis under visible light conditions (Scheme 24).^40^ Employing either NiCl_2_(DME)[^*t*Bu^tpy] or NiCl_2_(DME)[TPA] (4 mol%) and an Ir–F photocatalyst (1 mol%), they devised four sets of conditions for methylating different substrate classes. These conditions involved using either DTBP or DCP (6 equiv.) as the peroxide methyl source while varying the acid additives (0.5 equiv.) and solvents. The authors obtained 30 substrates in low to moderate yields ranging from 28–61%, including 5 instances of late-stage functionalisation. The reaction generally lacked enantioselectivity and, in certain cases, exhibited less site-selectivity, resulting in a mixture of diastereomeric and regioisomeric ratios. The remaining mass balance primarily comprised incomplete conversion as well as di- and multi-methylation. Depending on the set of conditions, several reactive functional groups were tolerated, including aryl and alkyl chlorides, aryl boronates and bromides, acidic O–H and N–H bonds, silyl-protected alcohols, quaternary alcohols, esters, benzisoxazole, lactam, ketones, primary sulfonamides, trifluoroacetamide, and carboxylic acid.

The authors further demonstrated that chemoselectivity can be controlled either by protonating or Boc-protecting basic amines with activated α-amino C(sp^3^)–H sites in the presence of competing benzylic reactive sites. Under innate selectivity, α-*N*-Boc C(sp^3^)–H sites preferentially undergo methylation in the presence of benzylic hydrogens. However, under modified conditions with a Brønsted acid present, the unprotected basic amine will protonate, thereby deactivating the α-amino C(sp^3^)–H sites. This causes a shift in chemoselectivity that favours benzylic C(sp^3^)–H methylation. Several examples were provided, yielding products in low to moderate yields ranging from 21–51%, including compounds such as safinamide, trimethoxybenzamide, cinacalcet, and homo-phenylalanine.

In the proposed mechanism (Scheme 24), the peroxide serves a dual role: it acts both as a source of the methyl radical through β-scission and facilitates hydrogen atom transfer (HAT) to generate the carbon radical necessary for cross-coupling. Initially, the methyl radical produced *via* β-scission coordinates with a Ni(i) species, forming a Ni(ii)–Me intermediate. Subsequently, this methyl radical on the Ni(ii) species reacts with the carbon radical, generated through HAT, to produce the desired product and regenerate the Ni(i) catalyst. Interestingly, despite the slightly acidic conditions, Minisci-type methyl addition to pyridine-containing substrates did not compete with C(sp^3^)–H methylation. By tuning specific reaction parameters tailored to different substrate classes, the authors demonstrated the potential of achieving a balance between the rates of hydrogen atom transfer (HAT) and methyl-radical formation.

**Scheme 25:**
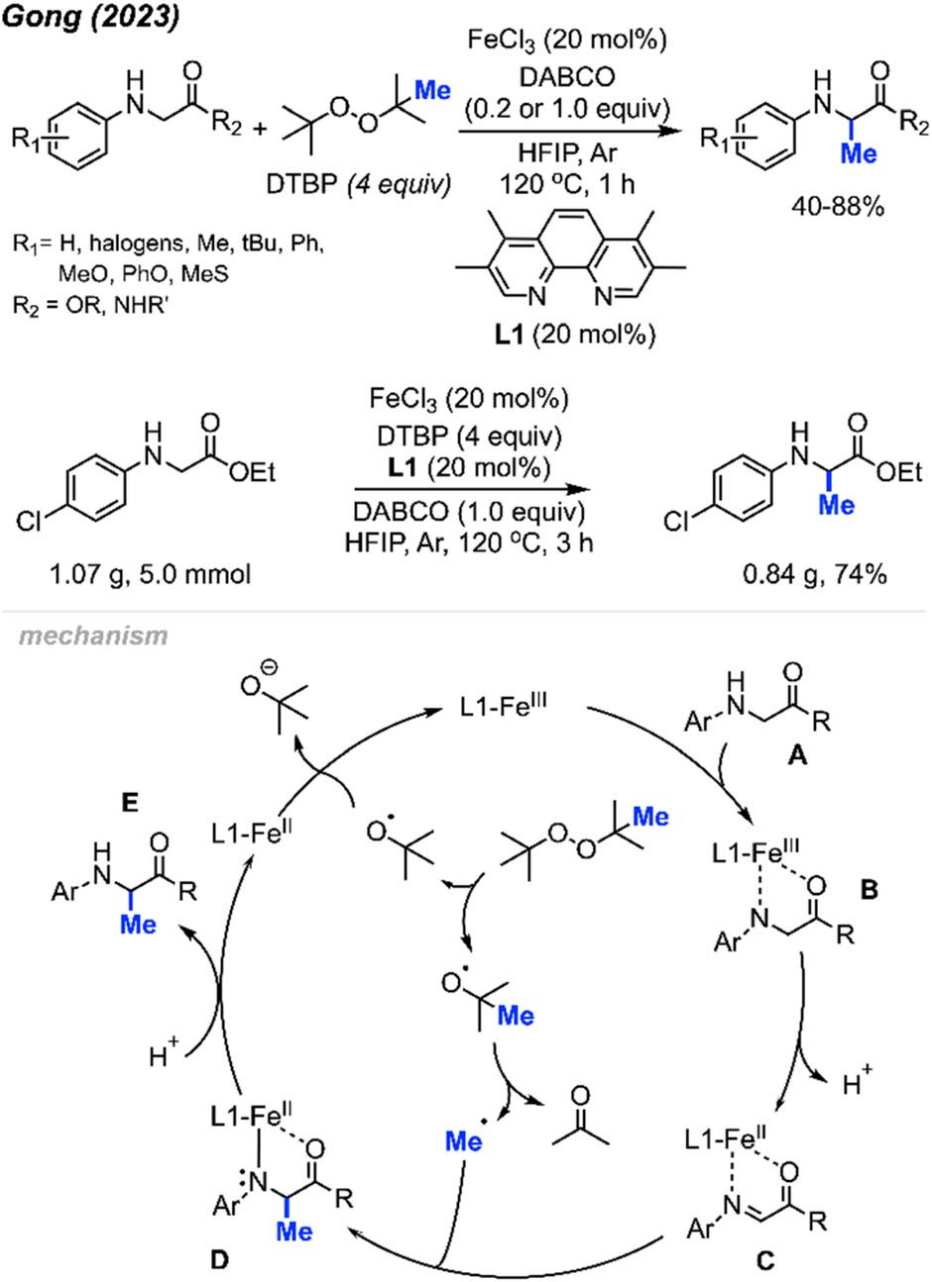
Iron-catalysed methylation of glycine and peptide derivatives. Scale-up of iron-catalysed methylation of glycine derivatives.

In 2023, Gong and co-workers reported the Fe(iii)-catalysed aliphatic C(sp^3^)–H methylation of glycine derivatives and peptides (Scheme 25).^41^ In the presence of DTBP, various *N*-aryl glycine esters and *N*-aryl glycine amides including some modified peptides can be selectively methylated, and 40–88% yields were achieved for 50 examples.

Using HFIP as the solvent drastically decreased the reaction time to 1 hour. The authors also examined the effects of *N*-substituents on glycine derivatives, and mono *N*-aryl substitution was found to be necessary. Several mono- and di-substituted glycine derivatives including N-Ph_2_, N-PhMe, N-HBoc, N-HFmoc, N-HCbz, and N-HMe did not work for this transformation. Showcasing the peroxide-mediated methylation of biologically relevant targets remains underexplored and has a wide application potential within pharmaceutical chemistry. To demonstrate the synthetic utility, the authors performed a gram scale reaction that yielded 74% isolated product within 3 hours under standard conditions (Scheme 25).

The proposed mechanism of this reaction involved an Fe(iii)/Fe(ii) catalytic cycle (Scheme 25). Firstly, *N*-aryl glycine substrate A undergoes ligand exchange with FeCl_3_ to form intermediate B. Subsequent intramolecular electron transfer and further deprotonation generates Fe(ii)–imine complex C. Concurrently, DTBP undergoes thermal homolysis to release the active *tert*-butoxy radical, which generates the methyl radical through β-scission. The methyl radical reacts with species C to form a coordinated complex D, which is protonated to produce the final product E. The released Fe(ii) intermediate is then oxidised by DTBP to regenerate the Fe(iii) catalyst.

### 2.2 Methylation of carboxylic acids

Methyl esters can be synthesised using various methods, including the Fisher esterification, the Steglich esterification, direct reaction of an acyl chloride with alcohols, and electrophilic methylation of carboxylic acid under basic conditions. Direct methylation of carboxylic acids by peroxides provides another option to obtain methyl ester derivatives. Until now, only two papers have been published regarding methyl esterification of carboxylic acids *via* peroxides.

**Scheme 26:**
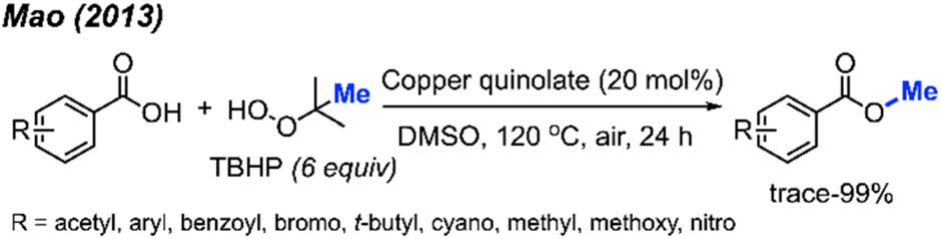
Copper-catalysed methyl esterification of carboxylic acids.

In 2013, Mao and co-workers reported that benzoic acid derivatives can be used to synthesise their corresponding benzoyl methyl esters *via* copper-catalysed cleavage of *tert*-butyl hydroperoxide (TBHP),^42^ providing 26 example reactions (Scheme 26). Benzoic acids with electron-withdrawing (nitro, cyano, benzoyl) or electron-donating groups (methoxy, methyl, *tert*-butyl) on the phenyl ring were all effective. Carboxylic acids on heterocyclic compounds such as indazole, thiophene, and furan were well tolerated, generating moderate to good yields.

**Scheme 27:**
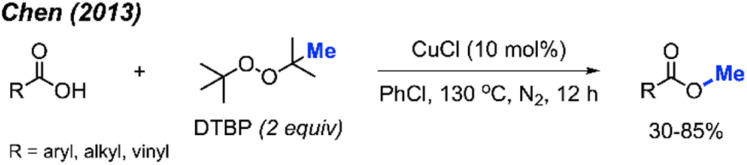
Copper-catalysed methyl esterification of carboxylic acids using DTBP.

In the same year, Chen and co-workers reported another methylation of carboxylic acids using peroxide catalysed by CuCl (Scheme 27).^43^ Both DCP and DTBP are good methylation reagents in the presence of CuCl (10 mol%), and 15 reaction examples were provided with 30–85% isolated yields. The authors found that alkyl acids gave higher yields than benzoic acids. Electron-withdrawn group at the *para*-position in benzoic acid strongly decreased the yields.

### 2.3 *S*-Methylation of sulfur containing compounds

Aryl methyl sulfides are a prevalent structural motif in bioactive natural products and therapeutic agents, while also serving as versatile synthetic intermediates for sulfoxides, sulfones, thiols and arenes. The synthesis of aryl methyl sulfides typically involves a coupling reaction between aryl halides and dimethyldisulfide, the reduction of methyl sulfoxides, or heteroatom-facilitated lithiation of aromatic carbon–hydrogen bonds followed by electrophilic substitution with dimethyldisulfide.

**Scheme 28:**
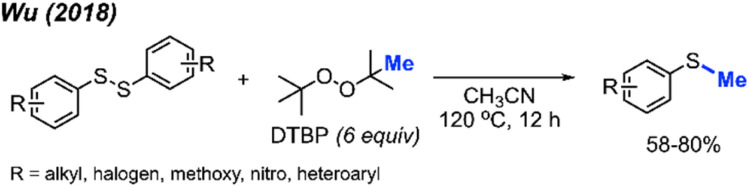
Metal-free sulfur methylation of diaryl disulfides with TDBP.

In 2018, Wu and co-workers reported a simple synthesis of aryl methyl sulfides using peroxides (Scheme 28).^44^ This reaction was run in neutral conditions, in absence of metal catalysts. Various functional groups (alkyl, halogen, methoxy, nitro, and heteroaryl) were tolerated in moderate to good yields. This represents a facile and environmentally friendly alternative to the traditional synthesis of aryl methyl sulfides.

**Scheme 29:**
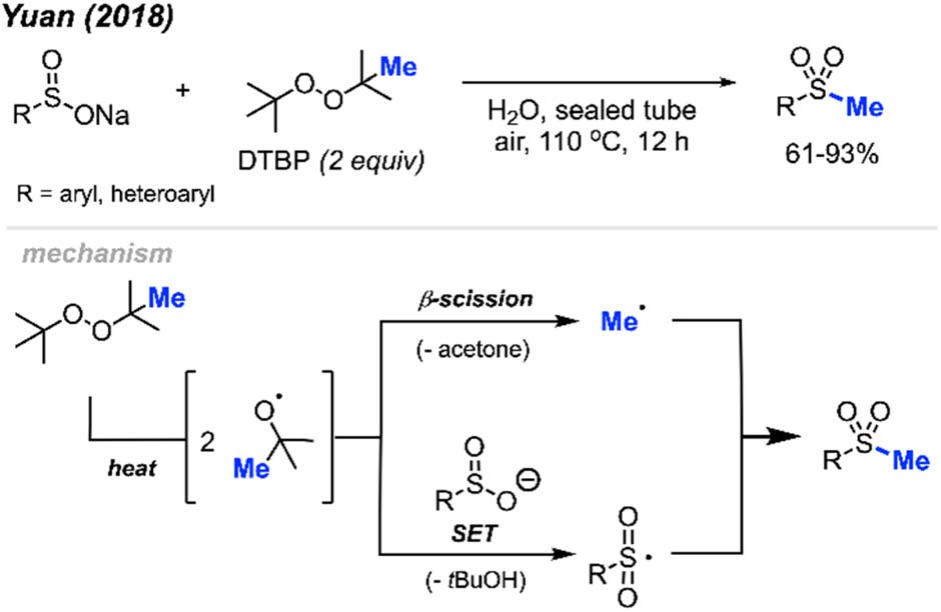
Metal-free sulfur methylation of diaryl disulfides with TDBP.

In 2018, Yuan and co-workers demonstrated a new method for synthesizing aryl methyl sulfones using sodium sulfinates and DTBP in water (Scheme 29).^45^ Under this metal-free system, products were obtained in 61–93% yields with good functional group tolerance, including halogens, ketones, thiophene, alkyl and methoxy groups. The authors suggest that the DTBP plays a dual role, first as an oxidant to generate the sulfinate radical and secondly to form the methyl radical that can react with the sulfinate radical.

**Scheme 30:**
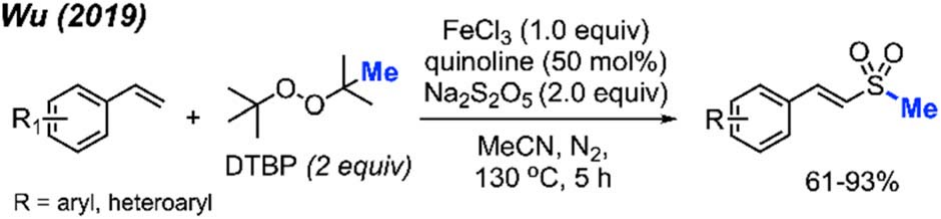
Iron-promoted C(sp^2^)–H methylsulfonylation of alkenes using DTBP.

In 2019, Wu and co-workers reported the direct C(sp^2^)–H methylsulfonylation of styrene derivatives using 1.0 equivalent of FeCl_3_ and 50 mol% quinoline under nitrogen at 130 °C (Scheme 30).^46^ DTBP was used as the methyl source and sodium metabisulfite (2.0 equiv.) was used to introduce the sulfone component. The product yields ranged from 61–93%, with functional group tolerance including halogens, aliphatic, and trifluoromethyl groups and one example with a vinyl thiophene methylsulfonylation. In the case of allylbenzene, prop-1-en-2-ylbenzene, allylbenzene and chalcone, no product was observed. This suggests that both the steric hindrance and electronics play a key role in this transformation.

### 2.4 Methylation of N–H bonds

The investigations of N–H bond methylation by peroxides are focused on amides (sulfonamides and methanamides), phosphonamides, phosphinamides, sulfoximines, and tetrazole compounds. Amides are widely present in nature and pharmaceutical compounds. The synthesis of amides commonly involves nucleophilic substitution of primary amides with alkyl halides under basic conditions, the reaction of acyl chlorides with amines, or a Steglich amide formation. Again, direct methylation of amide by peroxides provides another avenue for the amide synthesis.

**Scheme 31:**
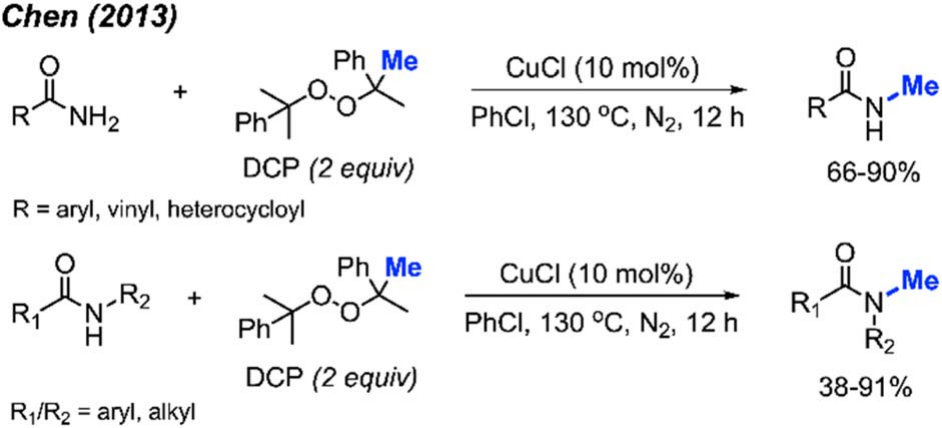
Copper-catalysed *N*-methylation of primary and secondary amides with DCP.

In 2013, Chen reported the *N*-methylation of various aromatic primary amides by DCP (2 equiv.) catalysed by CuCl (10 mol%) at 130 °C in PhCl as the solvent (Scheme 31).^43^ Both electron-donating groups (methyl, *tert*-butyl, methoxy) and electron-withdrawing groups (halogen, nitro, trifluoromethyl) on the phenyl ring worked well under these conditions. Heteroaryl amides (thiophene and furan) as well as cinnamamide were also successfully methylated. Secondary amides can be further *N*-methylated under the same conditions. Both aromatic amides and aliphatic amides afforded good yields (38–91%), including lactams.

**Scheme 32:**
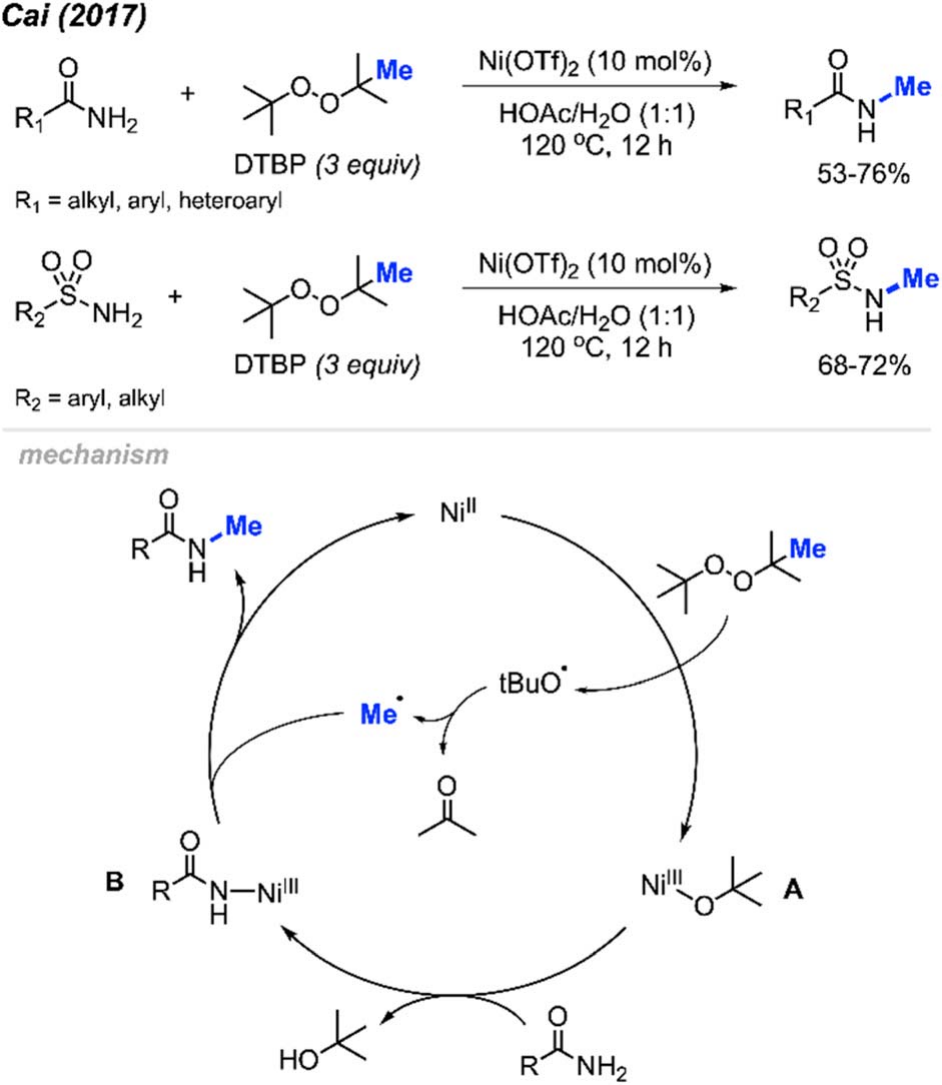
Nickel-catalysed *N*-methylation of amide and sulfonamides using DTBP.

In 2017, Cai reported a nickel-catalysed *N*-methylation of amides using peroxides (Scheme 32).^19^ Both amides and sulfonamides containing various substituents including methyl, methoxy, halogens, and nitro provided good yields under these reaction conditions. A total of 20 examples afforded 53–76% yields. Notably, *N*-methylation of maleimide, phthalimide, lactams, nicotinamide and indole were all successful. Lactams showed higher reactivity than linear alkyl amides.

**Scheme 33:**
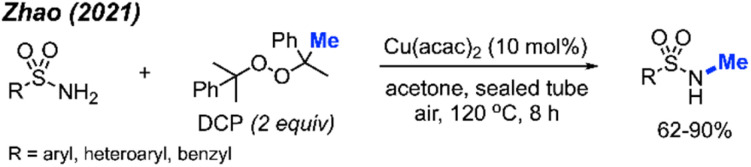
Copper-catalysed *N*-methylation of sulfonamides using DCP.

In 2021, Zhao and co-workers showed that methylation of sulfonamides can also be achieved with copper catalysis (Scheme 33).^47^ In this reaction, 10 mol% Cu(acac)_2_ is used in combination with DCP as the methyl source in acetone at 120 °C to obtain 21 product in yields ranging from 62–90%. Notably, the reaction demonstrated broad functional group tolerance, including halogens, methoxy, alkyl, trifluoromethyl, nitro, cyano, pyridine, quinoline, and thiophene. The authors also provided one example to show that the reaction is applicable to benzylamide.

Each of the *N*-methylation reactions undergo similar mechanisms (Scheme 32). Highlighted in Scheme 32 is the proposed mechanism from Cai and co-workers^19^. Initially, thermal homolysis of DTBP produces two alkoxy radicals, which then undergo β-scission to form the methyl radical and the corresponding ketone. Concurrently, DTBP oxidises Ni(ii) to the Ni(iii) alkoxide intermediate A. Metathesis of this intermediate with the amide substrate generates intermediate B and releases *t*BuOH. Intermediate B reacts with the methyl radical to form the *N*-methylated product while regenerating the Ni(ii) catalyst.

**Scheme 34:**
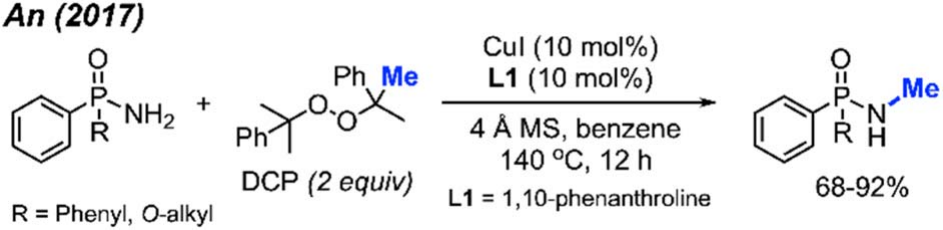
Copper-catalysed *N*-methylation of phosphonamides and phosphinamides.

Phosphonamides and phosphinamides are typical amino-fused organophosphorus compounds widely used in pharmaceutical and pesticide applications. An and co-workers first developed the *N*-methylation of phosphonamides and phosphinamides by peroxides *via* Cu-catalysis in 2017 (Scheme 34).^48^ DCP proved to be the best *N*-methylation reagent for these substrates, with 13 reaction examples presented. This is a novel and effective synthetic method toward *N*-methyl phosphonamides and phosphinamides.

**Scheme 35:**
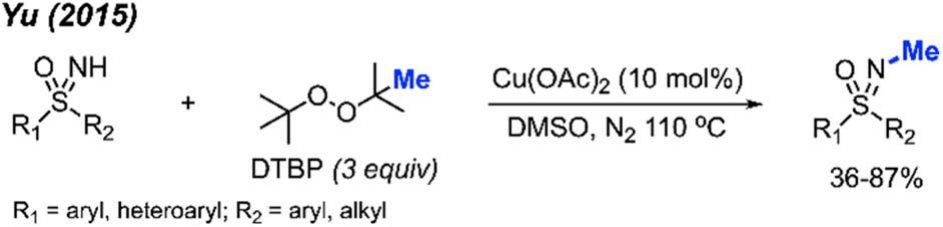
Copper-catalysed *N*-methylation of sulfoximines with DTBP.

In 2015, Yu reported the *N*-methylation of sulfoximines by DTBP catalysed by Cu(OAc)_2_ in DMSO at 110 °C (Scheme 35).^49^ A total of 15 reaction examples were provided with 36–87% isolated yields. Various functional groups (methyl, halogen, phenyl, methoxy, acetyl, and ester) on the phenyl ring were tolerated during the reaction. *S*,*S*-Diarylsulfoximines gave higher yields than *S*-aryl-*S*-alkyl sulfoximines. This reaction was conducted under neutral conditions without the use of acids and bases.

**Scheme 36:**
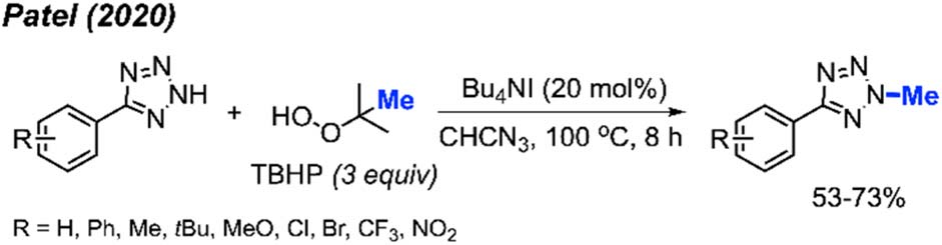
Metal-free *N*-methylation of aryl tetrazoles using TBHP.

Tetrazole-based *N*-heterocyclic compounds are widely used in biological, pharmaceutical, and material sciences. Tetrazoles have been used in agricultural applications including ligands herbicides and fungicides. In 2020, Patel and co-workers developed a direct *N*-methylation method of aryl tetrazoles using peroxides catalysed by Bu_4_NI (Scheme 36).^50^ In this metal-free reaction, TBHP was found to be the best methylation reagent. The authors were also able to methylate 1-methyl-1*H*-benzo[*d*][1,2,3]triazole and 2-methylbenzo[*d*]isothiazol-3(2*H*)-one 1,1-dioxide in 42% and 68% respectively. Notably, the reaction worked well in the presence of ketones on the substrate; however, aldehydes tended to undergo side reactions with peroxide radicals. Overall, electron-withdrawing groups on the aryl ring afforded slightly higher yields than electron-donating groups.

## 3 Methylation and other functionalisation

### 3.1 Methylative difunctionalisation of alkenes

**Scheme 37:**
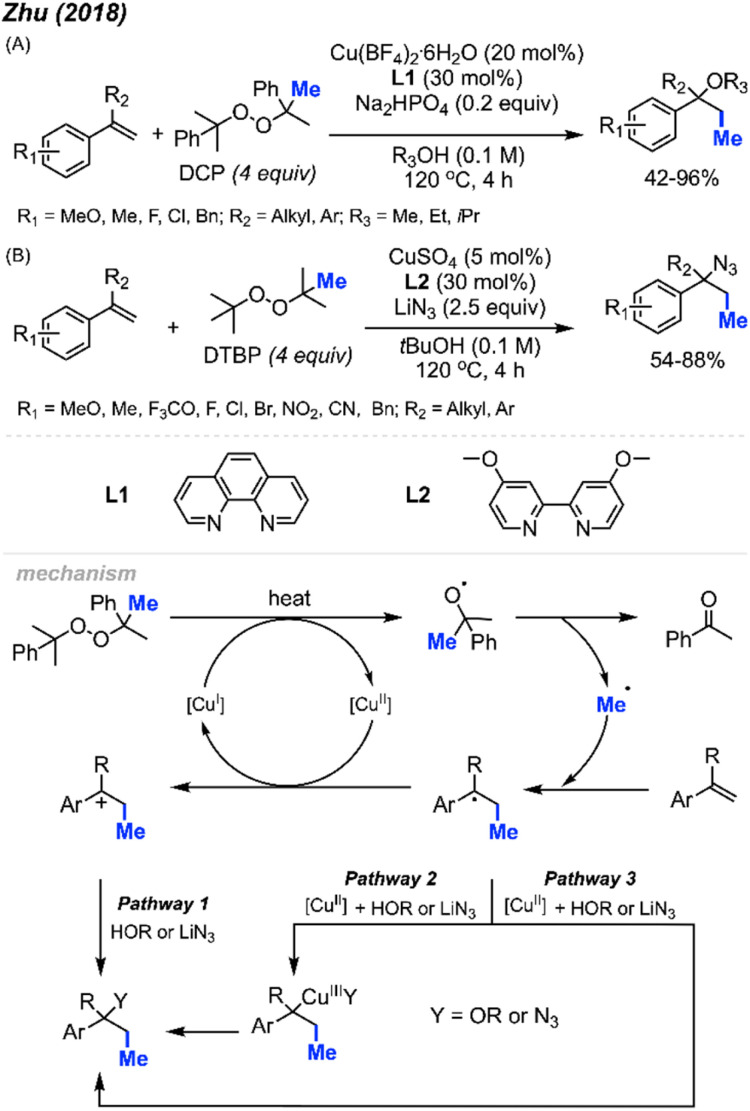
Copper-catalysed methylative difunctionalisation of alkenes. (A) Optimised reaction conditions using the solvent as the nucleophile, (B) optimised reaction conditions using LiN_3_ as the nucleophile.

In 2018, Zhu and co-workers were able to achieve methylative difunctionalisation of alkenyl C(sp^2^)–H bonds using copper catalysis (Scheme 37).^51^ Two sets of conditions were devised, one in which the solvent acts as the nucleophile (Scheme 37A) and the second in which the nucleophile is LiN_3_ (Scheme 37B). Both conditions afforded good overall yields, with similar functional group tolerance including methyl, methoxy, and halogens. In the case of the 1,2-azido methylation, the reaction also tolerated cyano, nitro, and trifluoromethoxy groups.

The mechanism (Scheme 37) is similar to that of Bao and co-coworkers.^36^ First, the homolytic cleavage of the peroxide is facilitated by Cu(i) and heat. Subsequent β-scission of the alkoxy radical generates the methyl radical, which reacts at the terminal end of the alkene to generate the more stable benzylic radical. The radical can then be oxidised by Cu(ii) to form the carbocation that can undergo nucleophilic attack (Pathway 1). Alternatively, the nucleophilic addition to the benzylic radical intermediate can either be achieved *via* a radical rebound to generated a Cu(iii) intermediate with the nucleophile and substrate which is then followed by reductive elimination (Pathway 2) or through a Cu-centred redox transfer process (Pathway 3).

**Scheme 38:**
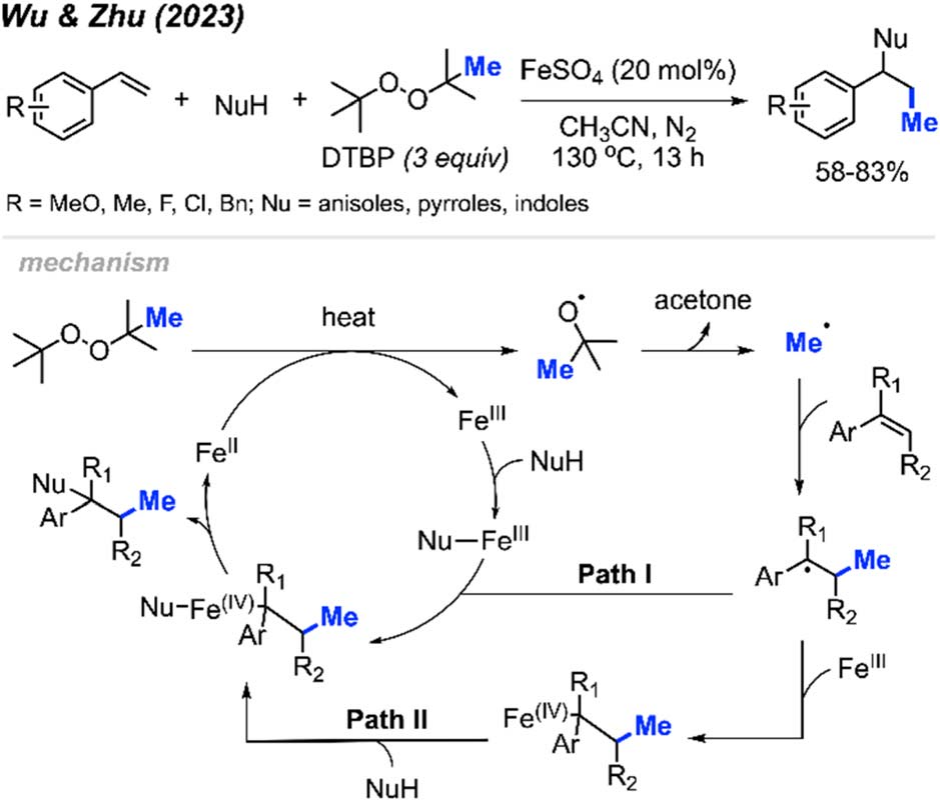
Iron-catalysed intermolecular 1,2-difunctionalisation of alkenes using DTBP.

More recently in 2023, Wu, Zhu, and co-workers were similarly able to achieve methylative difunctionalisation of alkenes using iron catalysis (Scheme 38).^52^ This was achieved with 20 mol% FeSO_4_ at an elevated temperature of 130 °C under nitrogen, using DTBP as a methyl source. In this case, the authors demonstrated the use of anisoles as nucleophiles for C–N bond formation, as well as pyrrole and indole nucleophiles that afforded C–C bond formation.

The authors proposed the mechanism shown in Scheme 32. Initially, Fe(ii) and heat promote the homolytic cleavage of DTBP, followed by β-scission to form *tert*-butoxide, acetone and the methyl radical. Upon addition of the methyl radical to the terminal end of the alkene, two possible pathways are proposed. Both pathways lead to an Fe(iv) intermediate containing the substrate and nucleophile, where subsequent reductive elimination afford the final product and regenerates Fe(ii).

### 3.2 Decarboxylative methylation

**Scheme 39:**
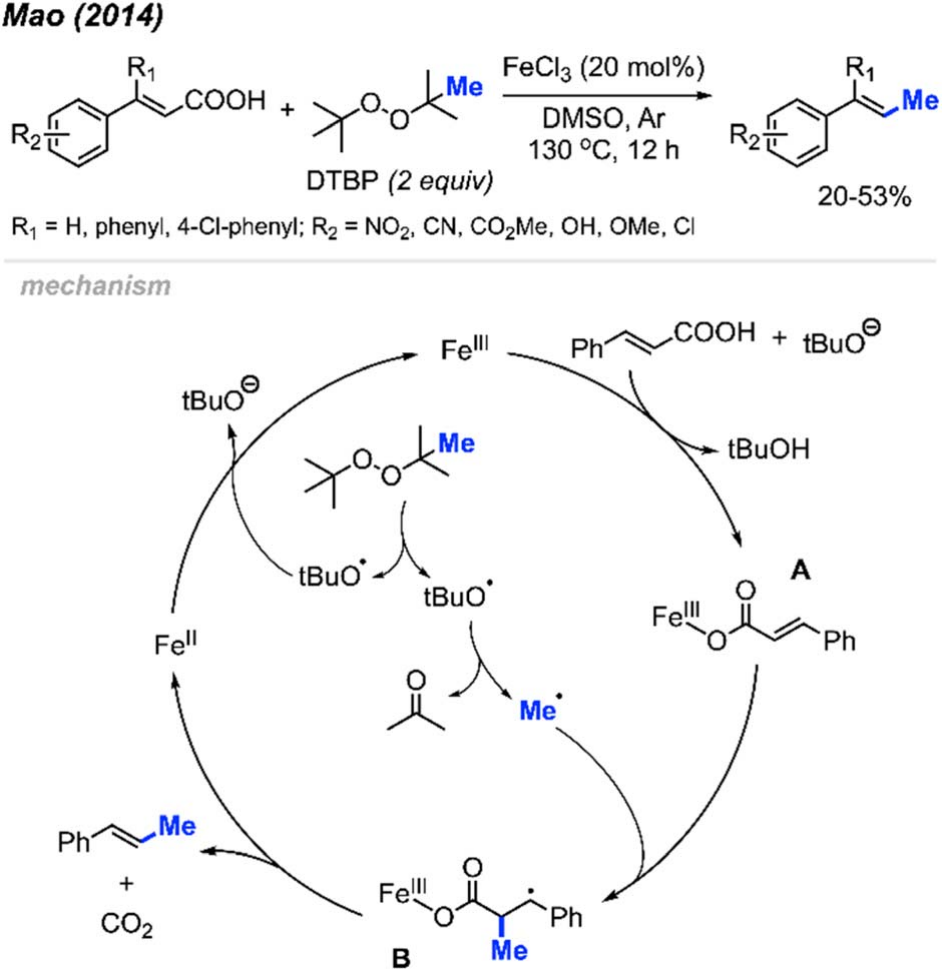
Iron-catalysed decarboxylative methylation of cinnamic acids.

In 2014, Mao and co-workers discovered the peroxide mediated decarbonylative methylation of cinnamic acid derivatives (Scheme 39).^53^ The reaction was achieved using FeCl_3_ catalyst and DTBP, where 12 reaction examples provided 20–53% yields with retained double bond configuration. Notably, β-substituted substrates such as β-phenyl cinnamic acid were tolerated.

Scheme 39 illustrates the proposed mechanism. The catalytic cycle initiates with the formation of a methyl radical released from a *tert*-butoxy radical. This radical subsequently adds to the α-position of the double bond on the ferric cinnamate intermediate A, formed through the reaction between cinnamic acid and ferric chloride, yielding intermediate B. Elimination of CO_2_ and Fe(ii) from intermediate B results in the final product. The regeneration of Fe(iii) occurs as Fe(ii) undergoes oxidation by DTBP.

### 3.3 Methylation and desulfonylation

**Scheme 40:**
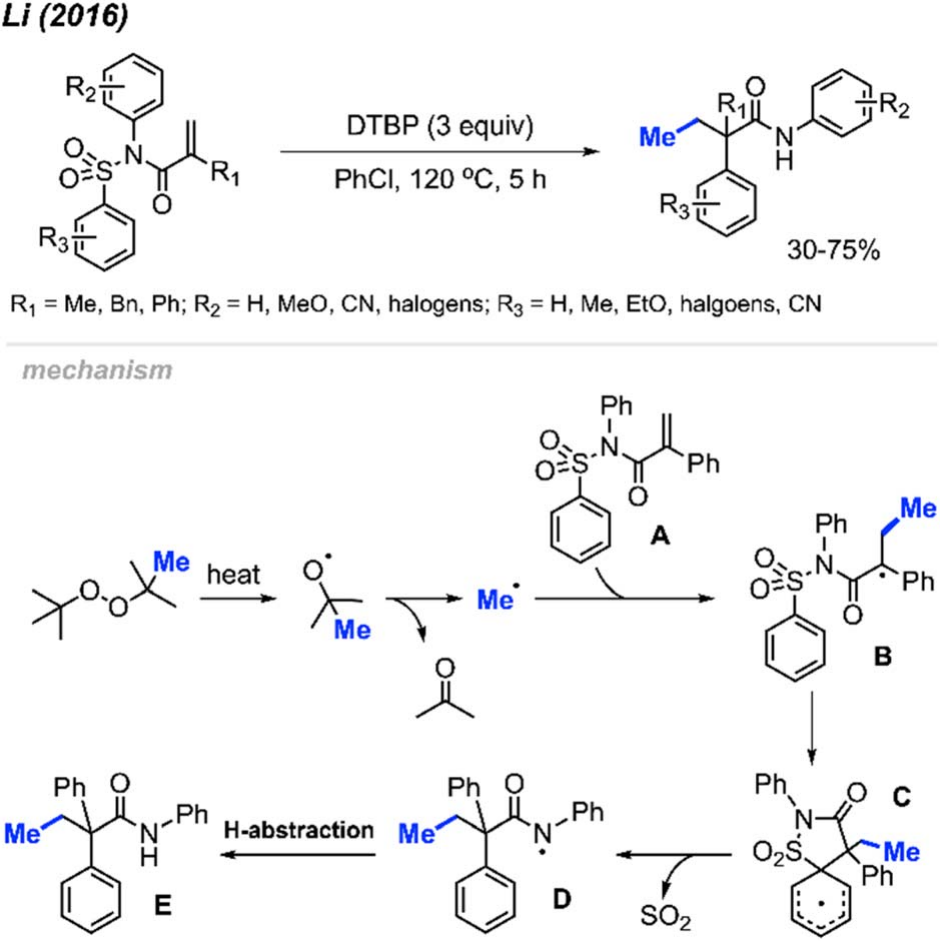
Oxidative 1,2-arylmethylation cascades of *N*-(arylsulfonyl)acryl-amides with DTBP.

In 2016, Li and co-workers reported a peroxide-promoted methylation/1,4-aryl migration/desulfonylation cascades of *N*-(arylsulfonyl)acrylamides, resulting in the formation of 2,2-disubstituted-*N*-arylbutanamides without the need for any metal catalyst (Scheme 40).^54^ The study presented 21 example reactions, showcasing yields ranging from 30–75%. Various peroxides, including DTBP, DCP, TBHP, and TBPB, exhibited reactivity in these conditions, with DTBP emerging as the most effective methylation reagent. The degree of reactivity was observed to vary with the position of the *N*-aryl *R*_2_ substituent, following the order: *para* > *meta* > *ortho*. This research introduces a facile and straightforward synthetic route for 2,2-disubstituted-*N*-arylbutanamides featuring an all-carbon quaternary stereocenter.

The authors proposed a possible mechanism outlined in Scheme 40. DTBP undergoes facile decomposition, yielding a methyl radical and acetone. Addition of the methyl radical across the alkene of the *N*-(arylsulfonyl)acrylamide A starting material produces the radical intermediate B. An intramolecular 5-*ipso*-cyclisation of intermediate B generates intermediate C, which subsequently undergoes desulfonylation to transform into amidyl radical species D. Finally, hydrogen abstraction by intermediate D leads to the formation of the desired product E.

**Scheme 41:**
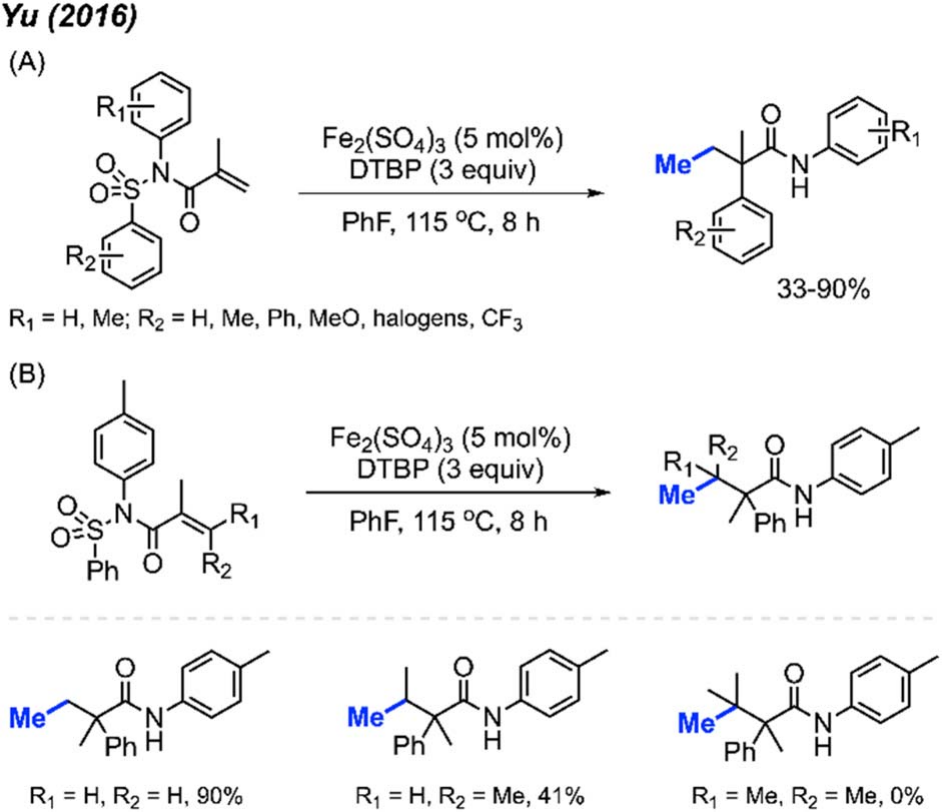
Iron-catalysed oxidative 1,2-arylmethylation cascades of *N*-(arylsulfonyl)acrylamides with DTBP. (A) Optimised reaction conditions, (B) product not observed when using di-methylated terminal alkenes.

In the same year, Yu and co-workers reported a similar investigation, finding that Fe_2_(SO_4_)_3_ could serve as a catalyst for the arylmethylation of sulfonyl acrylamides in the presence of DTBP (Scheme 41A).^55^ A total of 22 reaction examples were presented, achieving up to 90% yields. Notably, the authors observed a substantial substituent effect on the terminal alkene of the starting material. When a single methyl substituent was present at the terminal alkene, the reaction yield decreased to 41%. Interestingly, employing a di-methylated terminal alkene resulted in no observable reaction (Scheme 41B).

### 3.4 Oxidation and methylation

**Scheme 42:**
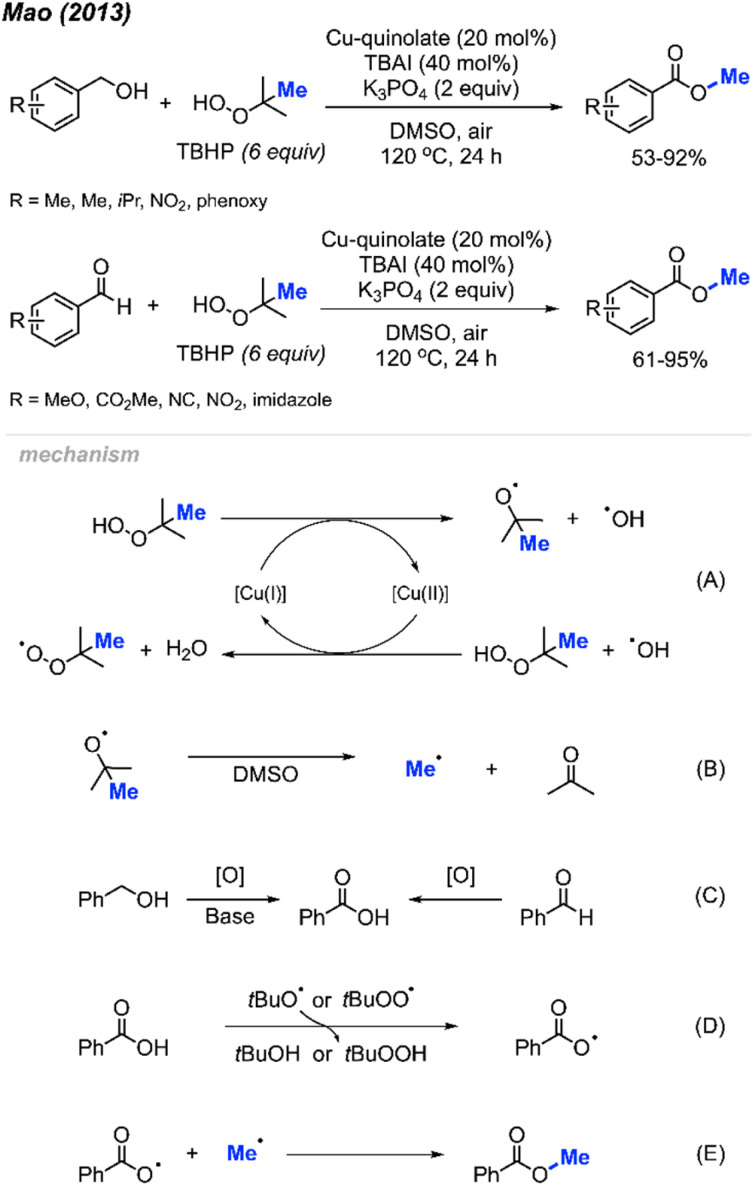
Copper-catalysed methyl esterification of benzyl alcohols and benzaldehydes.

In 2013, Mao and co-workers reported that benzyl alcohols and aldehydes can be oxidised and subsequently *O*-methylated in a one-pot synthesis of the corresponding benzoyl methyl esters using TBHP when catalysed by Cu(ii)-quinolate (Scheme 42).^42^ For these two reactions, both electron-withdrawing groups and electron-donating groups on the phenyl rings provided good yields. For the benzyl alcohol reactions, K_3_PO_4_ (2 equiv.) was used as base to assist the oxidation process. Tetrabutylammonium iodide (TBAI) was used as an additive since it was observed to accelerate the methyl esterification process.

**Scheme 43:**
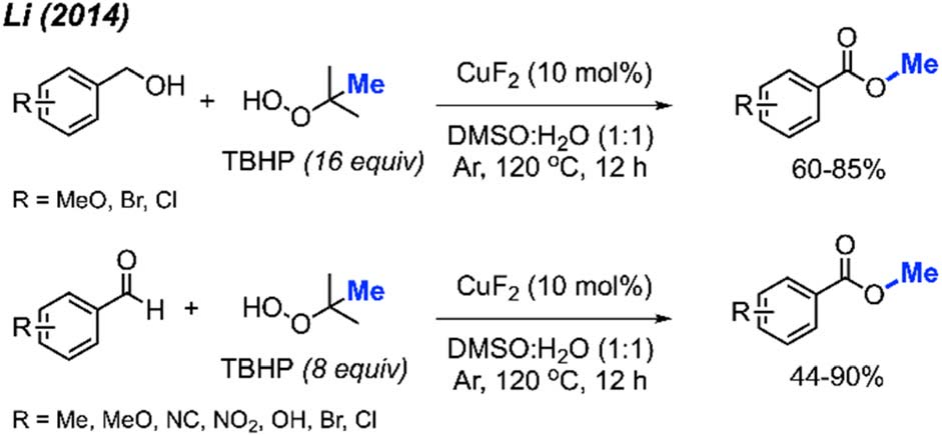
Copper-catalysed methyl esterification of benzyl alcohols and benzaldehydes.

In 2014, Li and co-workers demonstrated the copper-catalysed methyl esterification of benzaldehydes and benzoic alcohols under similar conditions (Scheme 43).^56^ In this case, a lower catalyst loading of 10 mol% CuF_2_ was used with a one to one mixture of DMSO and water as the solvent. Reaction yields ranged from 44–90%, with functional group tolerance including halogens, cyano, methoxy, nitro, methyl, and hydroxyl groups. Notably, thiophene-2-carbaldehyde was successfully esterified at 90% yield.

Both sets of authors proposed similar reaction mechanisms, as shown in Scheme 42. First, cleavage of TBHP to produce a *tert*-butoxy radical and hydroxide anion is facilitated by the coupled oxidation of Cu(i) to Cu(ii). The *tert*-butoxy radical further decomposes to yield the methyl radical and acetone. The Cu(ii) subsequently facilitates the conversion of another TBHP and a hydroxide anion into a *tert*-butylperoxy radical and water, thereby reducing back to Cu(i). Concurrently, the benzylic alcohol or aryl aldehyde is oxidised by the peroxide to the corresponding carboxylic acid with the help of base for the former. Hydrogen atom abstraction of the resulting carboxylic acid by the *tert*-butoxy or *tert*-butylperoxy radical then generates the acyloxy radical. Finally, the acyloxy radical reacts with the methyl radical to form the desired methyl ester.

**Scheme 44:**
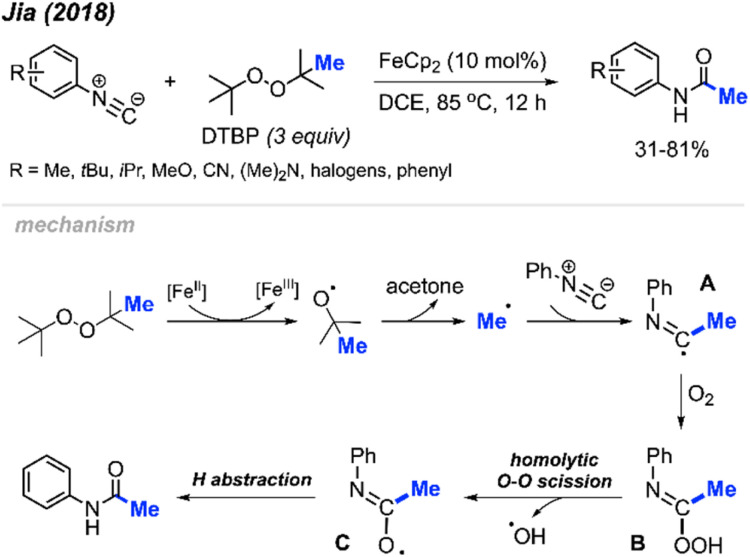
Iron-catalysed methylation and oxidation of aryl isocyanides.

In 2018, Jia and co-workers reported the peroxide facilitated methylation and oxidation of aromatic isocyanides to produce *N*-arylacetamide compounds (Scheme 44).^57^ This was achieved in the presence of FeCp_2_ (10 mol%) catalyst and DTBP, affording moderate to good yields (31–81%). Aromatic isocyanides containing various functional groups were well tolerated; however, aliphatic isocyanides such as *tert*-butyl, cyclohexyl, *n*-butyl, and benzyl isocyanides were not successful. As shown in Scheme 44, the authors propose a mechanism in which metal-assisted cleavage of DTBP generates a methyl radical. This radical then reacts with the isocyanide to form intermediate A, which reacts with oxygen to produce intermediate B. Homolytic cleavage of the peroxide then forms intermediate C, which undergoes hydrogen abstraction to yield the final product.

### 3.5 Methylation and cyclisation

#### 3.5.1 Methylation and mono-cyclisation

In 2014, three research groups independently reported similar difunctionalisations of alkenes, employing radical methylation through methyl-containing peroxides. This oxidative methylation and cyclisation introduced a novel synthetic approach towards oxindole scaffold production, a class of molecules prevalent in natural products and pharmaceuticals.

**Scheme 45:**
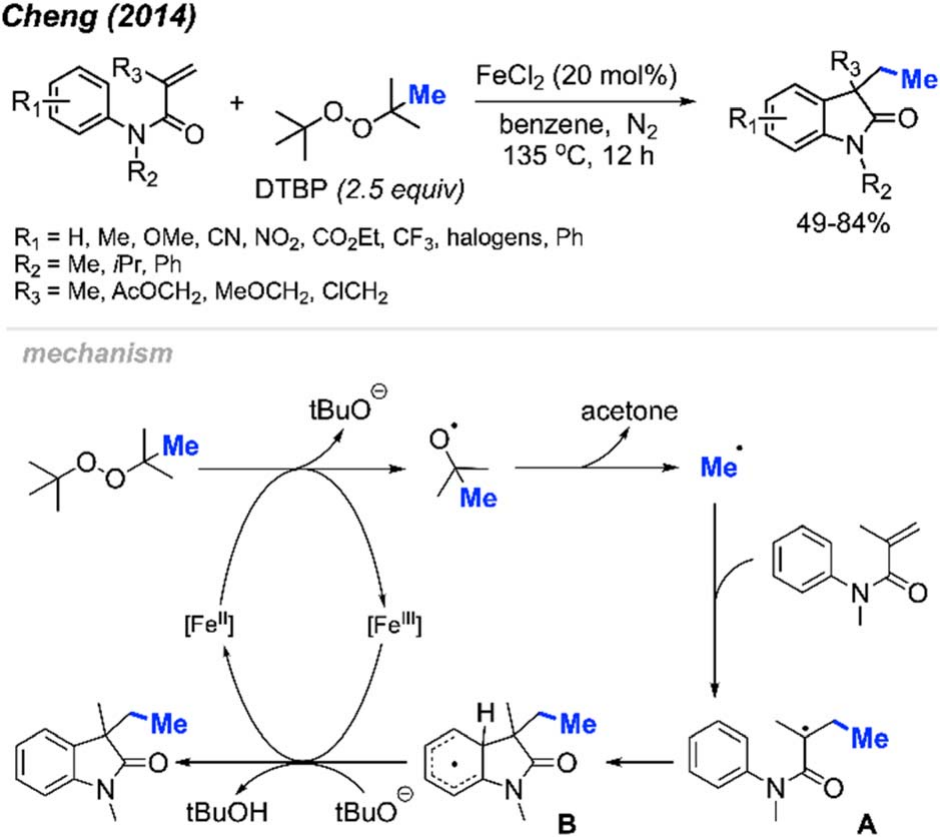
Iron-catalysed sequential difunctionalisation of alkenes with DTBP.

Initially, Cheng and co-workers observed that FeCl_2_ facilitates the carbomethylation of arylacrylamides using DTBP, resulting in the synthesis of 3-ethyl-3-substituted indolin-2-one in moderate to good yields (Scheme 45).^58^ The reaction exhibited tolerance towards various functional groups, such as cyano, nitro, ethoxy carbonyl, halogen, and trifluoromethyl groups. The researchers provided approximately 30 examples of successful reactions.

The reaction mechanism is outlined in Scheme 45. Initially, the reaction between DTBP with Fe(ii) produces *tert*-butoxy radicals, leading to the generation of acetone, a *tert*-butoxy anion, and a methyl radical. The methyl radical then reacts with the starting material, resulting in the formation of intermediate A. Subsequently, intermediate A undergoes intramolecular cyclisation with the phenyl ring, yielding intermediate B. The Fe(iii) then oxidises intermediate B to form an intermediate cation, where subsequent deprotonation by the *tert*-butoxy anion restores aromaticity to generate the final product.

**Scheme 46:**
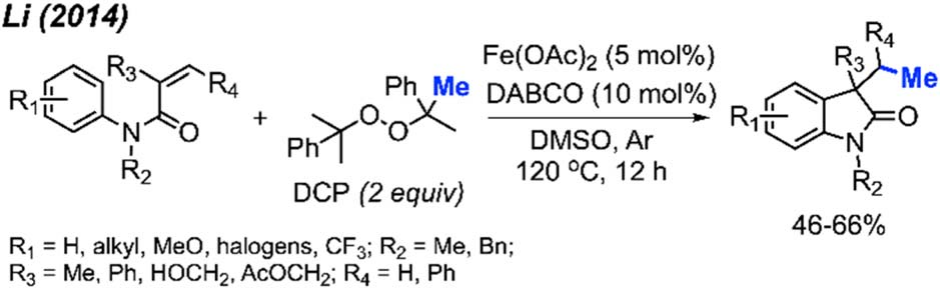
Sequential difunctionalisation of alkenes with DCP.

Li and co-workers presented a study with analogous findings (Scheme 46).^59^ The authors screened various peroxides, including TBHP, DTBP, DCP, and cumyl hydroperoxide. Among these, DCP emerged as the most effective reagent for the methylation/cyclisation reaction. Various functional groups demonstrated good compatibility under the optimised conditions. The reactivity order of substituents on the phenyl ring was observed to be *para* > *meta* > *ortho*. The authors also determined that the *R*_2_ substituent on the nitrogen atom cannot be a hydrogen, since the free NH acrylamide exhibited no reactivity under these conditions. The study presented 16 examples, showcasing yields ranging from 46–66%.

**Scheme 47:**
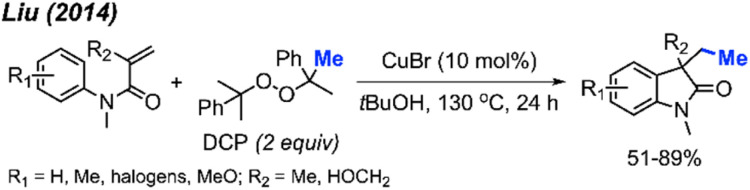
Sequential difunctionalisation of alkenes with DCP.

In the same year, Liu and co-workers reported the third example for the methylation/cyclisation reaction promoted by DCP (Scheme 47).^60^ The authors opted for CuBr as the catalyst, and, notably, hydroxyl groups and halogens are resilient under these reaction conditions. A total of 12 examples were presented containing various functional groups, yielding 51–89% yields.

**Scheme 48:**
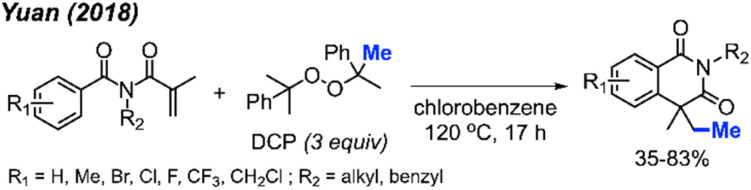
Metal-free sequential difunctionalisation of alkenes with DCP.

In 2018, Yuan and co-workers showed that it was possible to achieve a metal-free methylative cyclisation of *N*-alkyl-*N*-methacryloylbenzamides to generate methylated isoquinoline-1,3-diones (Scheme 48).^61^ The reaction was performed in chlorobenzene at 120 °C, using DCP as the methyl source. The overall reaction yields ranged from 35–83%, with a total of 17 examples. Tolerance to halogens, trifluoromethyl, chloromethyl, methyl, and *tert*-butyl functional groups was observed. It was also observed that product formation was favoured with electron-donating groups and hindered in the presence of electron-withdrawing groups. In the case of *p*-methoxy substitution, a mixture of products was obtained that reflected reactions occurring at both the *ortho*- and *meta*-positions, likely due to increased reactivity of the benzene ring.

**Scheme 49:**
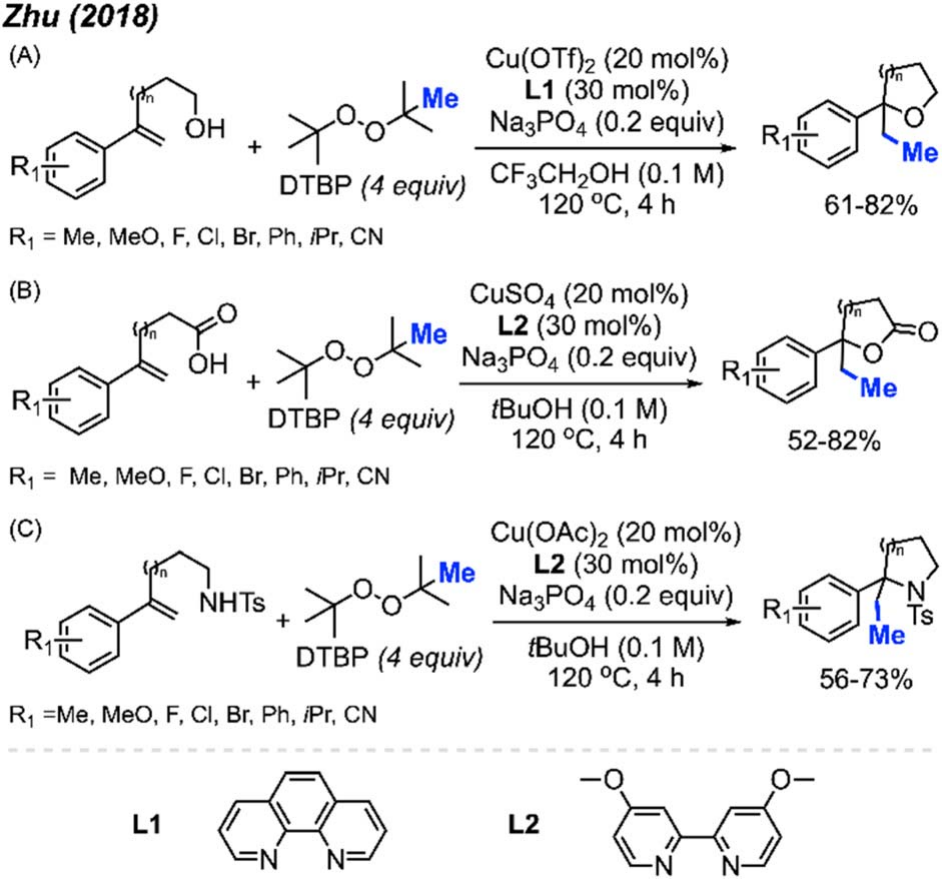
Copper-catalysed methylative cycloetherification, lactonisation, and cycloamination using DTBP. (A) Optimised reaction conditions for methylative cycloetherification, (B) optimised reaction conditions for methylative lactonisation, (C) optimised reaction conditions for methylative cycloamination.

In 2018, Zhu and co-workers utilised the difunctionalisation of styrenes to achieve methylative cycloetherification, lactonisation, and cycloamination (Scheme 49).^51^ In each case, the copper pre-catalyst and conditions were tuned to facilitate the specific cyclisation, with DTBP used as the methyl source in all cases. The product yields across the three cyclisation reactions ranged from 52–82%, with functional groups tolerance towards halogens, methoxy, methyl, iso-propyl, and cyano groups. Notably, a regioselective methylative lactonisation of (*E*)-4-phenylbut-3-enoic acid to afford the 4,5-*trans*-disubstituted γ-lactone as one diastereomer in a 41% yield was demonstrated. In this case, the mechanism follows that of the methylative difunctionalisation in Scheme 30, with the nucleophile directly on the starting substrate to facilitate cyclisation.

**Scheme 50:**
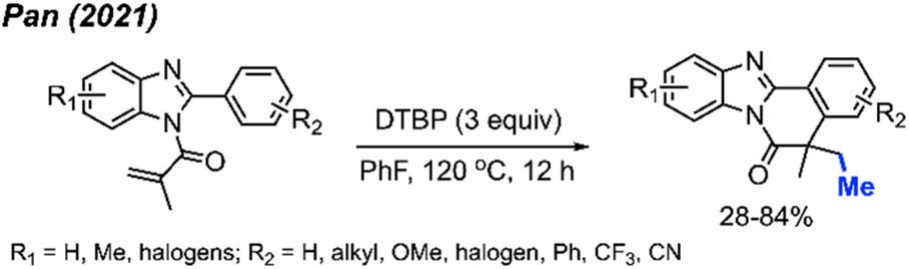
Synthesis of benzimidazo[2,1-*a*]isoquinoline-6(5*H*)-ones promotred by DTBP.

In 2021, Pan and co-workers extended their exploration, introducing a novel peroxide-driven methylation/cyclisation method for the synthesis of benzimidazo[2,1-*a*]isoquinoline-6(5*H*)-ones. Starting from *N*-methyacryl-2-phenylbenzimidazoles and DTBP, this approach yielded the corresponding products in good yields, all achieved without the need for any metal catalyst (Scheme 50).^62^ The study showcased 30 reaction examples, demonstrating yields ranging from 28–84%. This research not only broadens the synthetic toolkit but also offers a new strategy for constructing benzimidazo-fused polycyclic scaffolds.

**Scheme 51:**
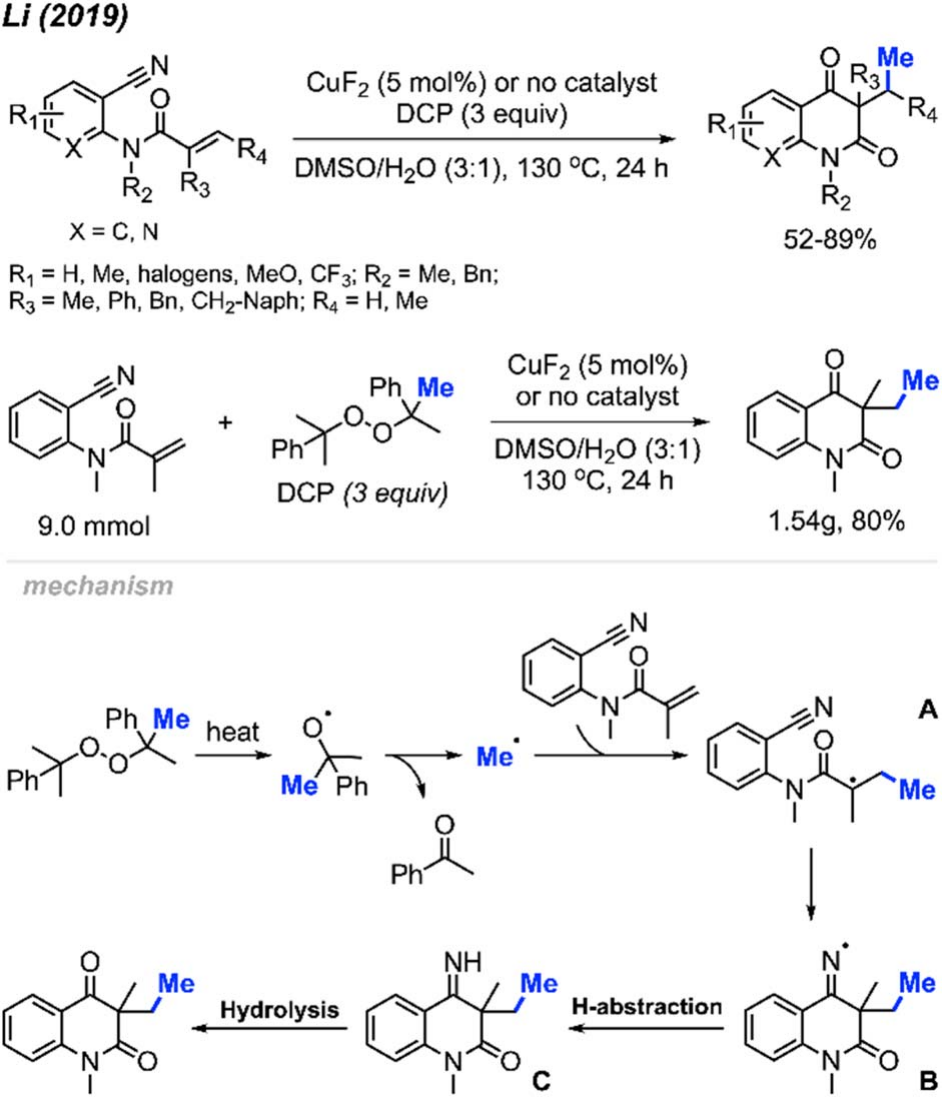
Copper-catalysed sequential difunctionalisation of *ortho*-cyanoarylacrylamides with DCP.

In 2019, Li and co-workers reported the cascade methylation and cyclisation of *ortho*-cyanoaryacrylamindes with DCP (Scheme 51).^63^ The reaction exhibited efficacy even in the absence of a transition metal catalyst, although higher yields were achieved in the presence of 5 mol% CuF_2_.

This transformation provides a simple and straightforward method for synthesizing methylated quinoline-2,4(1*H*,3*H*)-dione compounds. Notably, a gram-scale reaction resulted in an 80% isolated yield, showcasing the application of the methylation/cyclisation procedure towards organic synthetic applications without sacrificing efficiency.

The reaction mechanism is illustrated in Scheme 51. Thermal homolytic cleavage of DCP generates the cumyloxyl radical, which then undergoes β-scission to yield the methyl radical and acetophenone. Subsequently, the methyl radical reacts with the acrylamide alkene to afford intermediate A. The radical intermediate A attacks the cyano group, leading to the generation of imine radical B. This imine radical then facilitates H-abstraction, resulting in the formation of imine C. Hydrolysis of imine C ultimately yields the final product. The presence of CuF_2_ was thought to either enhance the acrylamide alkene reactivity, or stabilise the radicals generated during the reaction.

**Scheme 52:**
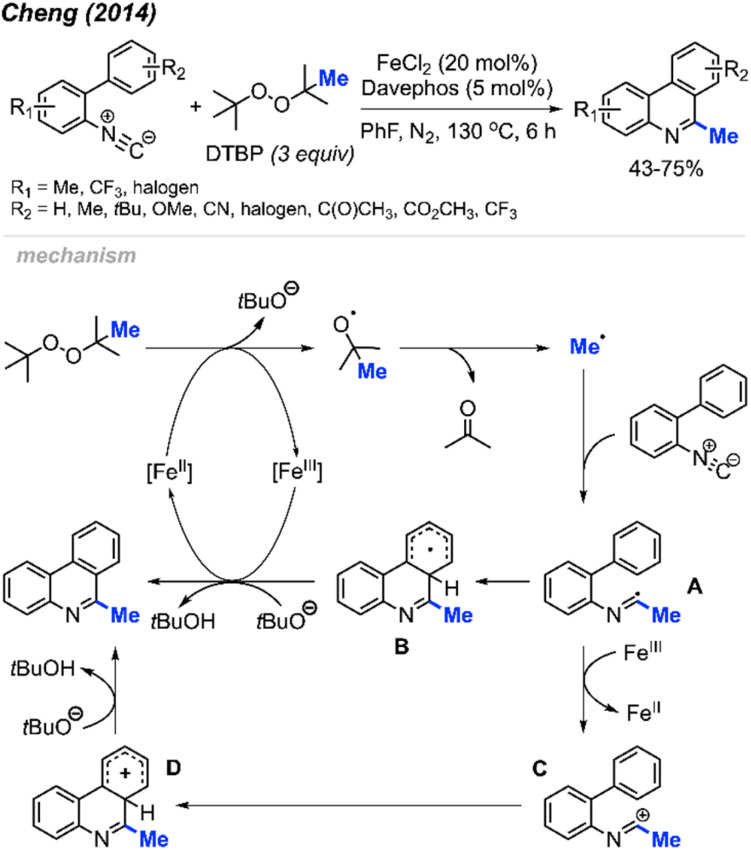
Iron-catalysed methylation and aromatisation of isonitrile promoted by DTBP.

Peroxides exhibit dual functionality by not only facilitating the methylation and cyclisation of *N*-arylacrylamides but also directing the methylation and intramolecular aromatisation of isonitriles. In 2014, Cheng and co-workers first reported the conversion of isonitrile into 6-methylphenanthridine derivatives utilizing DTBP and an Fe(ii) catalyst (Scheme 52).^64^ The reaction was tolerant of various functional groups, including fluoro, chloro, acetyl, methoxycarbonyl, cyano, and trifluoromethyl groups. Interestingly, no clear substituent effect was observed to impact the reaction efficiency.

The proposed reaction mechanism is illustrated in Scheme 52. Initially, Fe(ii)-assisted homolytic cleavage of DTBP generates the *tert*-butoxy radical, which then decomposes to the methyl radical and acetone. The methyl radical subsequently adds to the initial isonitrile substrate, forming intermediate A. Intramolecular cyclisation of intermediate A leads to intermediate B. Finally, Fe(iii) oxidises intermediate B to form an intermediate cation, which subsequently get deprotonated by *tert*-butoxide to form the final product. An alternative pathway involves single electron transfer (SET) between Fe(iii) and intermediate A, yielding cationic intermediate C. Subsequent aromatic electrophilic substitution (S_E_Ar) produces intermediate D, which undergoes proton loss to form the final product.

**Scheme 53:**
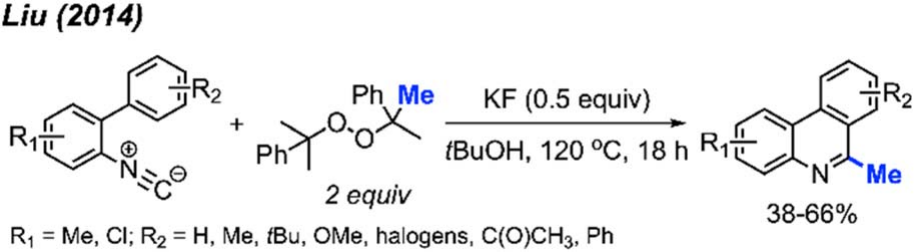
Methylation and aromatisation of isonitrile promoted by DCP.

Almost at the same time, Liu and co-workers reported a comparable study, revealing that DCP could promote the metal-free transformation of isocyanide into 6-methylphenanthridine compounds (Scheme 53).^60^ In this case, potassium fluoride was found to efficiently promote the reaction, although it was also functional in the absence of the KF promoter in lower yields.

#### 3.5.2 Methylation and bicyclisation

**Scheme 54:**
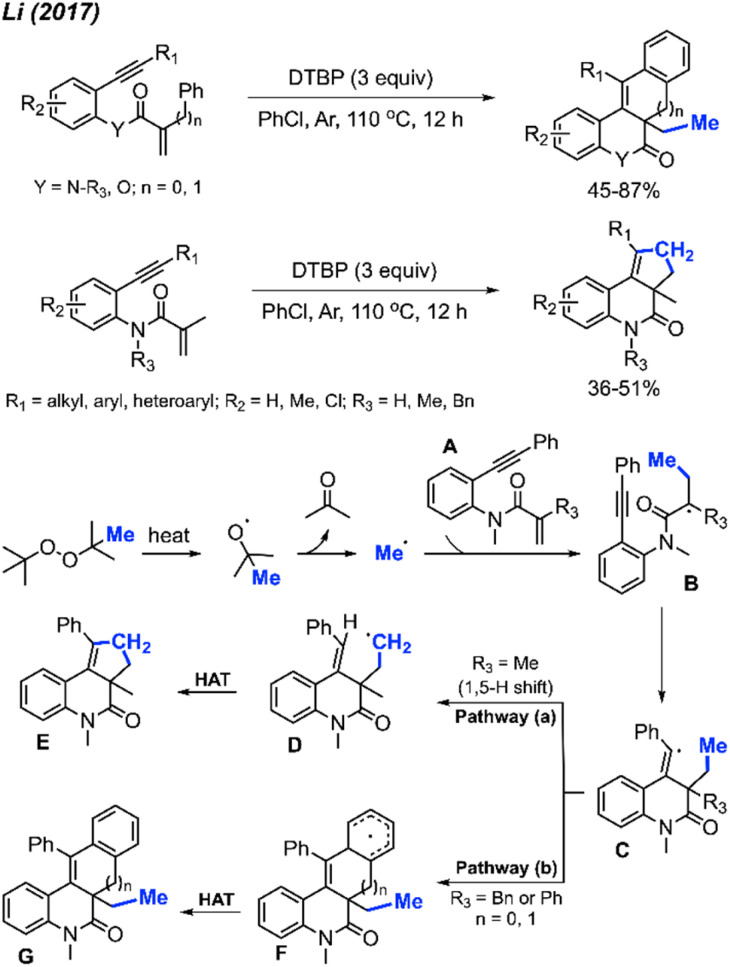
Metal-free annulation cascades of 1,7-enynes using DTBP.

Until now, only two research papers have explored peroxide-promoted methylation and bicyclisation, and notably, neither of them utilised any transition metal catalysts. In 2017, Li and co-workers developed an oxidative radical cascade methylation and bicyclisation protocol of *ortho*-alkynearyl acrylates and acrylamides with DTBP serving as the methylation reagent (Scheme 54).^65^ A total of 25 1*H*-cyclopenta[*c*]quinolone and benzo[*j*]phenanthridine compounds were synthesised in 36–87% yields.

Several peroxides, including DTBP, TBHP, TBPH, and DCP, exhibited efficacy in this reaction, with DTBP identified as the optimal methylation reagent. Among the solvents tested, PhCl proved to be the most suitable. The proposed reaction mechanism is depicted in Scheme 54. Under heat, DTBP generates a methyl radical and acetone. The methyl radical adds to the alkene on the starting material A to produce intermediate B. Subsequently, the radical intermediate B undergoes intramolecular cyclisation, forming the vinyl radical intermediate C. The fate of intermediate C diverges based on the nature of the substituents on the starting material. Pathway (a) involves an intramolecular 1,5-hydrogen shift, yielding the new alkyl radical intermediate D when *R*_3_ is a methyl group. The process concludes with a hydrogen atom transfer (HAT) of intermediate D, facilitated by an oxidant, to produce the final product E. In contrast, Pathway (b) entails a direct intramolecular cyclisation with the aryl ring, leading to the aryl radical intermediate F when *R*_3_ is a benzyl or phenyl group. Intermediate F proceeds through HAT to produce final product G.

**Scheme 55:**
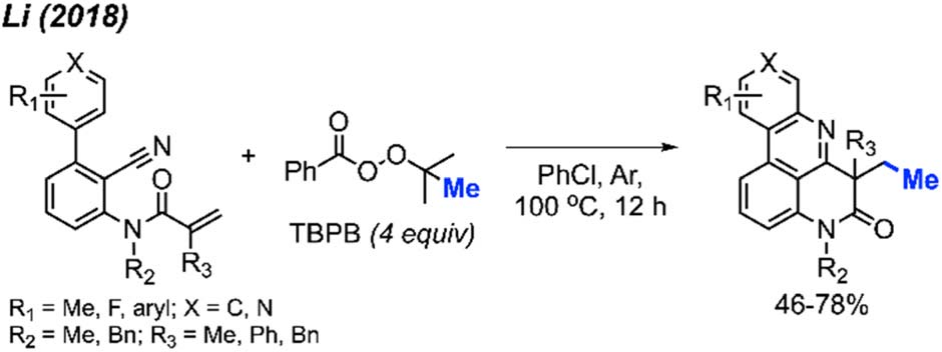
Metal-free radical bicyclisation of 2-cyanoaryl acrylamides with TBPB.

In 2018, Li and co-workers demonstrated another example of a metal-free bicyclisation to access methyl substituted polyheterocycles (Scheme 55).^66^ The authors provided 8 examples with yields ranging from 46–78%, including methoxy, fluoro and extended aryl groups. A variety of *N*-methyl groups were also tolerated, including methyl and benzyl groups, however, the secondary amide was not tolerated. When *R*_1_ was a *meta*-substituted methyl group, a mixture of two regioselective products was obtained. The reaction follows a similar mechanism to that show in Scheme 54.

**Scheme 56:**
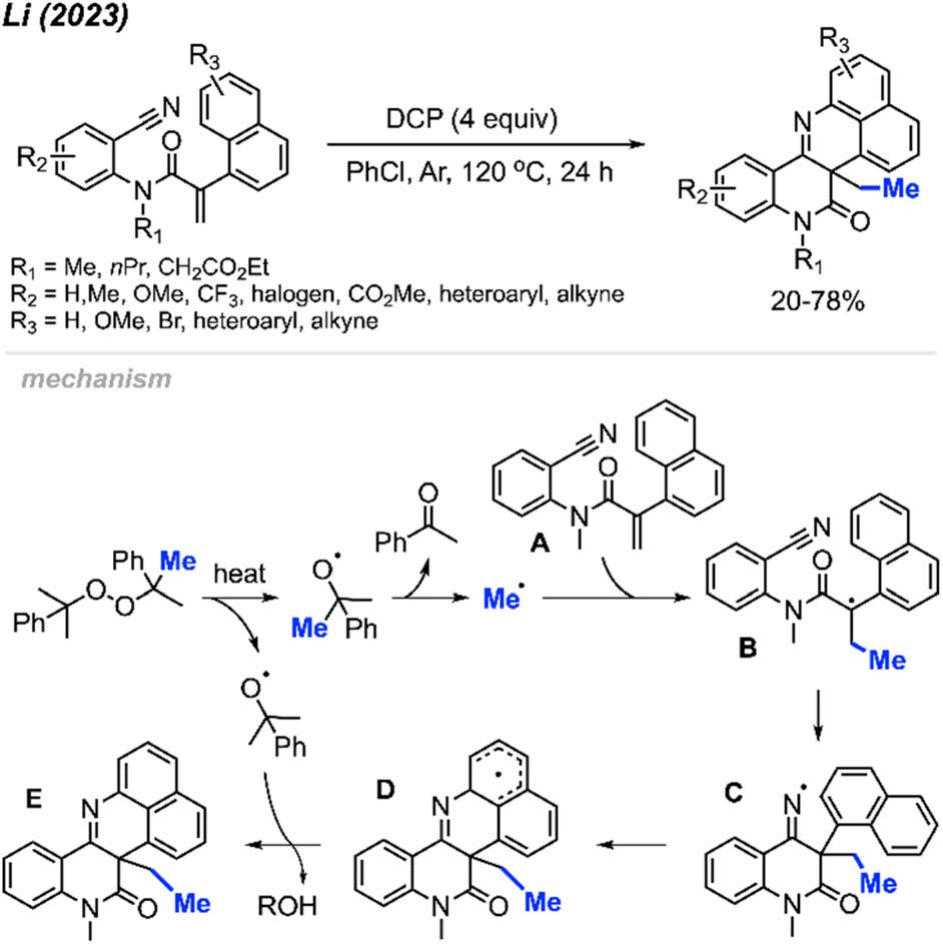
Metal-free radical bicyclisation of 2-cyanoaryl acrylamides with DCP.

In 2023, Li and co-workers extended the exploration of their metal-free *ortho*-cyanoaryl acrylamide radical bicyclisation reaction, introducing a new method towards benzo[*h*]naphtho[1,8-*bc*][1,6]naphthyridine scaffold synthesis (Scheme 56).^67^ A total of 22 example compounds were successfully synthesised in 20–78% yields. Tolerance of various functional groups was observed, including methyl, methoxy, methyl ester, halogens and trifluoromethyl groups on the phenyl ring. Moreover, pyridine, thiophene, and alkyne-substituted phenylacrylamides also resulted in satisfactory yields. Notably, the authors also found that acrylamides with *N*-benzyl or a free N–H were not suitable for this transformation.

The reaction mechanism is depicted in Scheme 56. When heated, homolytic cleavage of DCP releases cumyloxyl radicals, leading to acetophenone and the methyl radical formation through β-scission. The methyl radical then adds to the alkene of acrylamide A, forming the carbon-centred radical intermediate B. Subsequent intramolecular cyclisation of intermediate B results in the formation of the imine radical C, which further cyclises with the naphthyl ring to produce intermediate D. Finally, the oxidative aromatisation of intermediate D culminates in the formation of the final polyheterocycle product.

### 3.6 Methylene formation *via* intermediate methylation

#### 3.6.1 Methylene formation and cyclisation

**Scheme 57:**
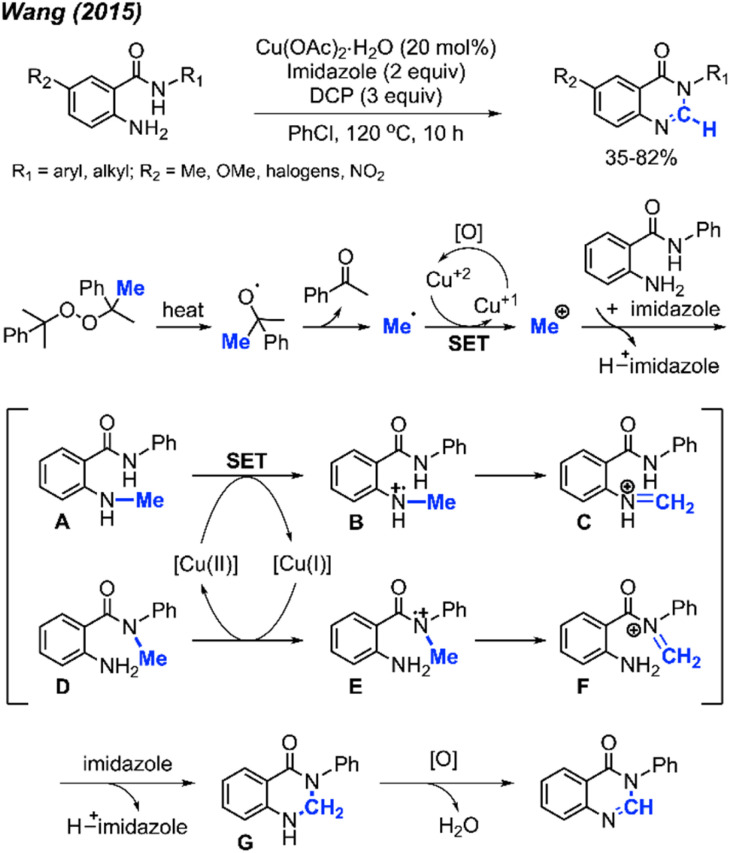
Copper-catalysed methylation and cyclisation towards the synthesis of quinazolinones promoted by DCP.

In 2015, Wang and co-workers reported a synthetic method for producing *N*-substituted quinazolinone derivatives, starting with anthranilamides and employing a Cu(ii) catalyst.^68^ DCP was the most effective methylation reagent in this context (Scheme 57). Notably, the study revealed that when the *R*_1_ functional group on anthranilamide was aromatic, the resulting products were obtained in higher yields (60–82%) compared to when *R*_1_ substituents were aliphatic (35–42%). Furthermore, elevated yields were achieved when *R*_1_ was a phenyl ring with electron-withdrawing group attached. The authors were also the first to confirm the presence of the active methyl radical, generated from the peroxide, through electron paramagnetic resonance (EPR).

Scheme 57 depicts the proposed reaction mechanism. The process initiates with the thermal decomposition of DCP, yielding two cumyloxyl radicals. A cumyloxyl radical further decomposes to form a methyl radical and acetophenone. The methyl radical is then converted into a methyl cation through a single electron transfer (SET) facilitated by Cu(ii). Subsequently, deprotonation of the starting material by imidazole base followed by its nucleophilic attack on the methyl cation affords either intermediate A or D. In the presence of Cu(ii), intermediate B can be derived from intermediate A. Following a hydrogen radical abstraction, intermediate C is formed. The amination product G is generated through an intramolecular nucleophilic attack within intermediate C, followed by deprotonation. Finally, intermediate G is oxidised to yield the final quinazolinone product.

**Scheme 58:**
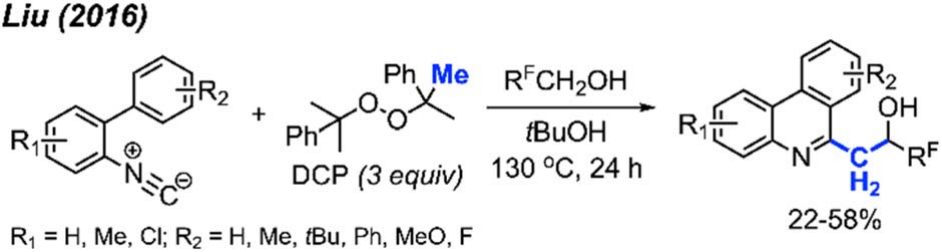
Metal-free methylation, aromatisation, and perfluoronated alcohol functionalisation cascade promoted by DCP.

Later in 2016, Liu and co-workers extended their previous work by coordinating a domino reaction to achieve methylation, aromatisation, and perfluorinated alcohol functionalisation under metal-free conditions (Scheme 58).^69^ The reaction was achieved at an elevated temperature of 130 °C using DCP as a methyl source. The authors demonstrated 17 examples in yields ranging from 22–58%, with functional groups including methyl, chloro, methoxy, and *tert*-butyl groups. A variety of polyfluorinated alcohols were also well tolerated. The reaction initially follows the same mechanism as Scheme 52. This is followed by phenanthridine isomerisation to afford the active enamine, which can subsequently react with the polyfluorinated alcohols to produce the final product.

#### 3.6.2 Methylene formation and amidation

**Scheme 59:**
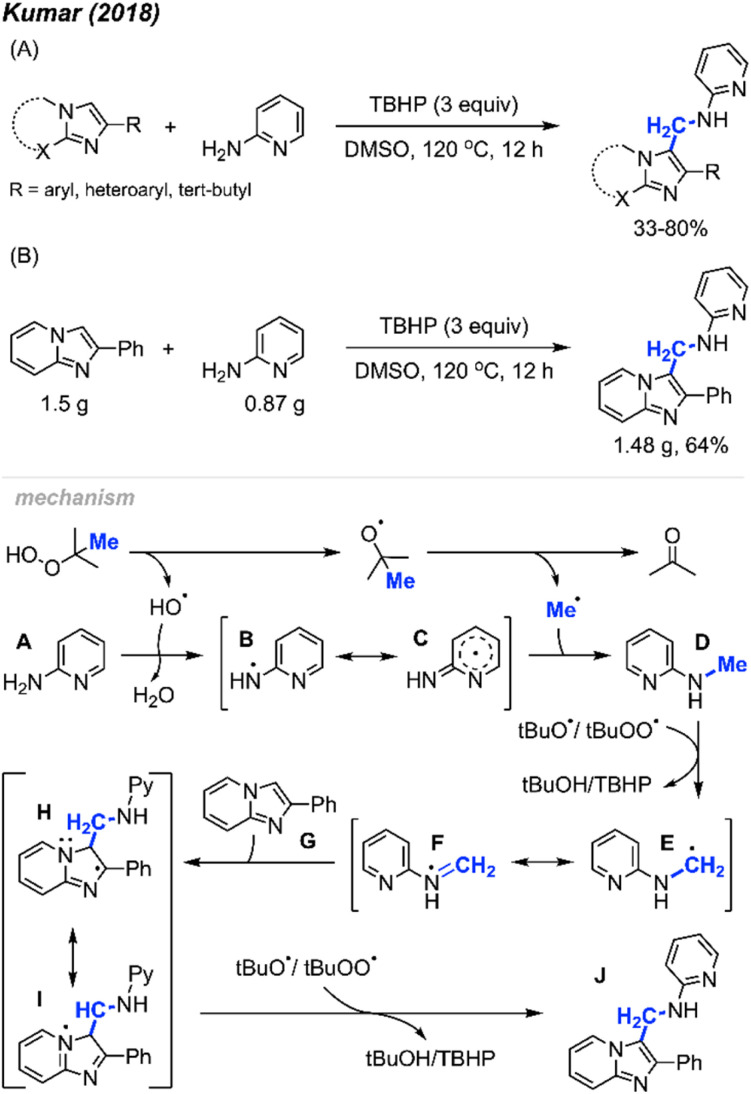
Metal-free synthesis of amino-methylated imidazo-heterocycles. (A) Optimised reaction conditions, (B) gram scale reaction.

In 2018, Kumar and co-workers described the regioselective synthesis of amino-methylated imidazo-hetercycle compounds promoted by peroxides (Scheme 59A).^70^ They found that TBHP could react with 2-aminopyridine, forming 2-methylaminopyridine, which then reacts with imidazole compounds to produce functionalised imidazo-heterocycles, assisted by TBHP. Notably, this reaction is metal-free, with DMSO serving as the solvent. The study provided 30 substrate examples, showcasing yields ranging from 33–80%. The reaction exhibited excellent tolerance of various functional groups, including methyl, methoxy, halogens, nitro, cyano, trifluoromethyl, and *tert*-butyl groups on the aromatic ring. Additionally, 2-aminopyridine, 2-aminopyrimidine and 2-aminopyrazine proved effective in this reaction. To assess the practical utility of this method, a gram-scale experiment was conducted, yielding 64% isolated product (Scheme 59B). This research introduced a new strategy towards imidazole-heterocycle synthesis, which are a distinct class of nitrogen heterocycles known for their diverse biological and photophysical properties.

The proposed reaction mechanism is illustrated in Scheme 59. TBHP undergoes homolytic cleavage, generating a *tert*-butoxy radical that then produces the methyl radical through β-scission. Subsequently, either a *tert*-butoxy, hydroxy, or *tert*-butylperoxy intermediate radical abstracts a hydrogen radical from 2-aminopyridine A, resulting in the formation of the aminyl radical intermediate B, which is stabilised by the pyridine ring. Intermediate B then reacts with the methyl radical to form intermediate D. Hydrogen atom abstraction from intermediate D yields the radical species E. The radical addition of E to the starting substrate G generates intermediate H, which, upon hydrogen abstraction, affords the desired product J.

### 3.7 Methylation and alkynylation of terminal alkenes

**Scheme 60:**
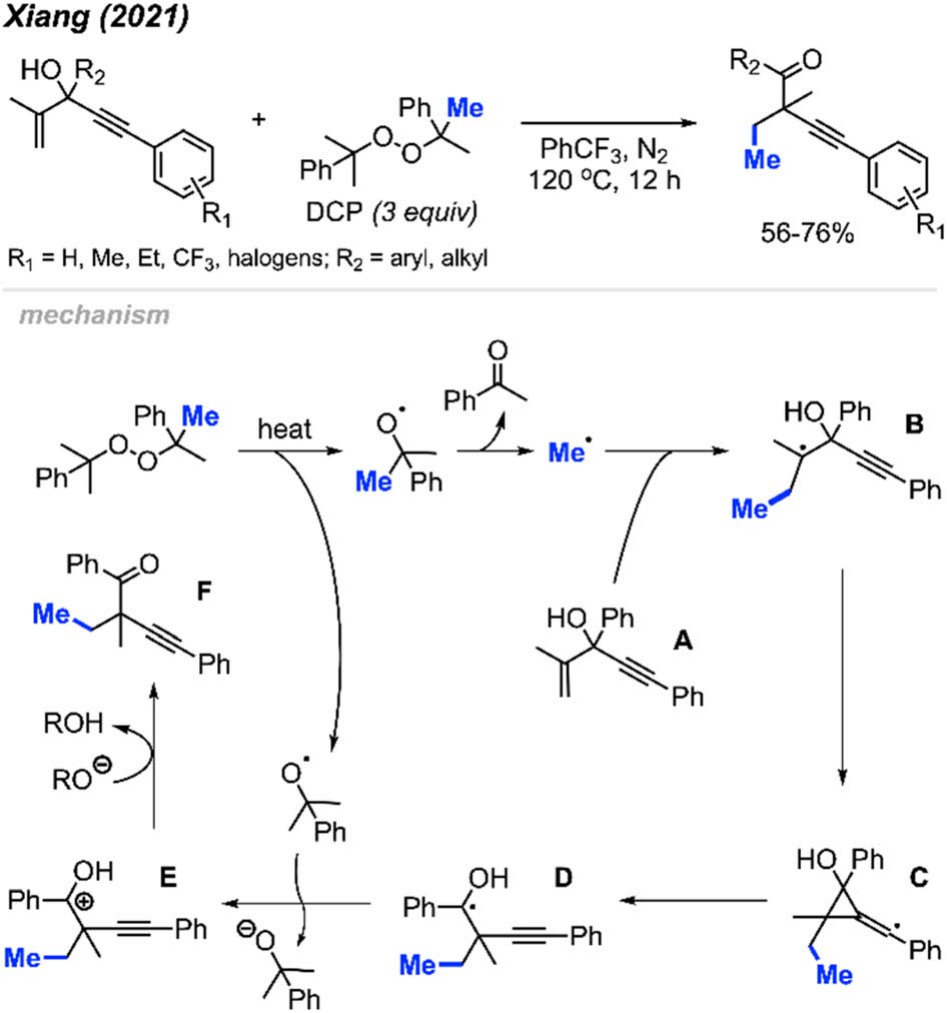
Metal-free methylation/alkynylation of terminal alkenes using DCP.

In 2021, Xiang and co-workers reported an oxidative, metal-free method for alkene methylation/alkynylation of 1,4-enyn-3-ols using DCP as the methyl source (Scheme 60).^71^ The 1,4-enyn-3-ol derivatives are reactive in PhCF_3_ at 120 °C, resulting in the synthesis of challenging quaternary-carbon containing but-3-yn-1-one products. DCP and DTBP were comparably effective as methylation reagents. The authors also found that the degree of reactivity varied with position of the *R*_1_ substituent on the alkynyl–aryl ring, following the sequence: *para* > *meta* > *ortho*. This study serves as another illustrative example of peroxides' utility as a methylation reagent within organic synthesis applications.

The authors proposed the mechanism depicted in Scheme 60. DCP thermally decomposes to generate a methyl radical and acetophenone. The methyl radical reacts with the starting material A, leading to the formation of radical intermediate B. This is followed by an unconventional, “anti-Baldwin” 3-*exo*-dig cyclisation and alkynyl migration, involving homolysis of the carbon–carbon bond, generating the hydroxyalkyl radical intermediate D. Single electron oxidation of intermediate D by a cumyloxyl radical produces the carbocation intermediate E. Finally, deprotonation of intermediate E yields the desired product F.

## 4 Conclusions

This review highlights significant advancements in the utilisation of peroxides as methylating reagents. Many of these innovative reactions operate without the need for transition metal catalysts, resulting in cleaner reactions, simpler workups, and enhanced suitability for late-stage functionalisation of biological compounds. Tandem processes have also been discovered, paving the way for more concise synthetic routes to generate useful products. Many of these reactions exhibit broad tolerance for various functionalities, encompassing both electron-donating and withdrawing groups. Particularly noteworthy is the inert nature of halogen substituents in this chemistry, providing an opportunity to use them as potential handles for further functionalisation. Insofar, the use of methyl-containing peroxides has emerged as a versatile pathway for the methylation of value-added products.

## Data availability

No primary research results, software or code have been included and no new data were generated or analysed as part of this review.

## Author contributions

Daliah Farajat and Yuhua Zhang prepared the draft, which was revised and edited by Professor Chao-Jun Li.

## Conflicts of interest

There are no conflicts to declare.
